# A dual role for ER-Golgi cargo receptor LMAN1 in supporting CSFV replication and restraining RLR signaling

**DOI:** 10.1128/jvi.00069-26

**Published:** 2026-03-03

**Authors:** Kailiang Han, Zhaoyu Chang, Ning Li, Dong Xiao, Mengzhao Song, Tao Wang, Kangkang Guo, Liang Zhang, Wen Deng

**Affiliations:** 1 College of Veterinary Medicine, Northwest A&F University 718173, Yangling, Shaanxi, People's Republic of China; 2 State Key Laboratory for Animal Disease Control and Prevention, College of Veterinary Medicine, Lanzhou University, Lanzhou Veterinary Research Institute, Chinese Academy of Agricultural Sciences 111658, Lanzhou, China; University of Kentucky College of Medicine, Lexington, Kentucky, USA

**Keywords:** classical swine fever virus (CSFV), lectin, mannose binding 1 (LMAN1), NS5B, RNA synthesis, RLR pathway

## Abstract

Classical swine fever virus (CSFV) establishes efficient infection by manipulating host factors and intracellular pathways to support viral replication. The host lectin mannose-binding 1 (LMAN1) is a cargo receptor that is involved in the assembly and morphogenesis of several RNA and DNA viruses. However, the function and potential mechanism of LMAN1 in CSFV infection remain elusive. Here, we investigated the LMAN1 expression pattern in CSFV-infected pigs and PK-15 cells, showing upregulation of the protein after CSFV infection. LMAN1 knockdown and overexpression further demonstrated that LMAN1 expression was required for efficient CSFV proliferation. Mechanistically, LMAN1 directly associates with the viral RNA-dependent RNA polymerase NS5B via its carbohydrate recognition domain (CRD), promoting efficient viral replication complex formation. Beyond its vital role in viral RNA replication, LMAN1 also functions as a negative regulator of antiviral signaling. Transcriptomic analyses revealed that LMAN1 deficiency hyperactivates the RIG-I-like receptor (RLR)-MAVS pathway, and further studies confirmed that loss of LMAN1 enhances IRF3 and NF-κB p65 phosphorylation, as well as interferon-β production in response to CSFV. MAVS knockdown reversed the interferon signaling hyperactivation caused by LMAN1 deficiency, and LMAN1 rescue experiments showed that the CRD domain is indispensable for suppression of interferon responses, revealing that LMAN1 modulates antiviral responses via the RLR-MAVS pathway relying on its CRD domain. Overall, our findings uncover LMAN1 as a previously unrecognized host determinant with dual function in CSFV replication and immune evasion, offering new insights into virus-host interactions and revealing a potential antiviral target.

**IMPORTANCE:**

Classical swine fever virus (CSFV) remains a major threat to global swine health, yet the host factors that the virus exploits to support virus infection and proliferation are not fully understood. Here, we identify the host cargo receptor lectin mannose-binding 1 (LMAN1) as a critical determinant of CSFV infection. LMAN1 directly binds the viral RNA-dependent RNA polymerase NS5B and is recruited to endoplasmic reticulum-derived replication membranes to promote efficient viral RNA synthesis. At the same time, LMAN1 suppresses RIG-I-like receptor signaling, limiting MAVS-mediated activation of IRF3, NF-κB, and downstream antiviral responses. Loss of LMAN1 disrupts CSFV replication complex formation and triggers robust antiviral signaling, revealing that the virus relies on LMAN1 for efficient replication and immune evasion. These findings uncover a novel dual function of a cargo receptor in promoting viral RNA synthesis while simultaneously restraining excessive innate immune responses, highlighting LMAN1 as a potential target for antiviral intervention.

## INTRODUCTION

Classical swine fever virus (CSFV), a member of the *Pestivirus* genus in the *Flaviviridae* family, causes a highly contagious disease in pigs characterized by hyperthermia, hemorrhagic syndromes, and persistent infection (1, 2). The virus carries a positive-sense (+) RNA genome that is translated into a single polyprotein, subsequently cleaved and processed into structural and non-structural (NS) proteins (3). The (+)RNA virus replication complexes (VRCs) are formed through extensive remodeling of host intracellular membranes (such as the endoplasmic reticulum [ER]), in conjunction with both viral and host factors. Within these microenvironments, NS5B functions as the RNA-dependent RNA polymerase that catalyzes viral RNA (vRNA) synthesis (4–6). The formation of VRCs is thought to provide an optimized platform for viral RNA synthesis while simultaneously shielding viral RNA and protein components from host antiviral surveillance mechanisms (6–10). Concurrently, the host innate immune system recognizes viral RNA through pattern recognition receptors (PRRs) such as RIG-I and MDA5, which signal through the adaptor MAVS to activate IRF3/7 and NF-κB, thereby inducing interferon (IFN)-β and a broad array of interferon-stimulated genes (ISGs). Increasing evidence suggests that RNA viruses exploit host trafficking machinery to facilitate replication (8).

Lectin mannose-binding 1 (LMAN1), also known as ER-Golgi intermediate compartment (ERGIC)-53, is a type I transmembrane lectin that localizes mainly to the ERGIC. Through its N-terminal carbohydrate recognition domain (CRD), LMAN1 functions as a selective cargo receptor for a subset of glycoproteins that cycle between the ER and the ERGIC/*cis*-Golgi (11, 12). Beyond its canonical role in the secretory pathway, accumulating evidence indicates that LMAN1 is broadly hijacked by pathogens to support infection. LMAN1 has been implicated in relation to several virus infections. LMAN1 acts as a binding partner for viral glycoproteins (GPs) and is a crucial cellular factor required for infectious Arenavirus, Coronavirus, and Filovirus production (13). Consistent with this role, LMAN1 governs virus productivity during lytic γ-herpesvirus infection (14) and can bind to the HIV glycoprotein Env (gp160/gp41) (15). In hepatitis B virus (HBV) infection, LMAN1 together with selective COPII components (Sec24A, Sec23B, and Sar1) drives virion export from the ER and engages pathogen-derived N-glycans on HBV (16). Conversely, LMAN1 knockdown diminishes viral entry, assembly, and release in hepatitis C virus (HCV) infection (17). LMAN1 also functions as a cell-surface receptor for house dust mite allergens that dampens NF-κB–dependent inflammatory signaling (18), underscoring its dual roles in viral infection and immunomodulation. Nevertheless, whether LMAN1 participates in the CSFV life cycle, how it might interact with CSFV proteins, and whether it influences innate immune signaling during CSFV infection remain unknown.

In our current study, we investigated the expression pattern of LMAN1 in *vivo* and in *vitro* during CSFV infection and revealed the impact of LMAN1 on CSFV infection via knockdown and overexpression. Interactions between LMAN1 and CSFV proteins were identified and mapped to subdomains. Furthermore, LMAN1 gene knockout and rescue cell lines were generated to explore its function and mechanism in CSFV infection. Transcriptome analyses were performed to reveal the biological processes regulated by LMAN1, followed by luciferase assays to explore its regulatory mechanism in the antiviral immune response. Through these studies, we identified LMAN1 as an essential host factor exploited by CSFV to promote viral replication and suppress innate immunity.

## MATERIALS AND METHODS

### Experimental animals

Six healthy 2-month-old piglets were cultured in isolators with filtered air under positive pressure in an SPF animal facility of the Experimental Animal Center of Northwest A&F University. Briefly, piglets were randomly divided into two groups, one challenged with 10^5^ 50% tissue culture infectious dose (TCID_50_) of CSFV and one inoculated with an equal volume of DMEM (19). Rectal temperatures were recorded each morning, and animals were observed daily for clinical signs. At 7 days post-infection (dpi), piglets from each group were euthanized, and designated samples were collected for immunohistochemistry, quantitative real-time PCR, and western blot analysis.

### Antibodies and reagents

The mouse polyclonal anti-CSFV E2 antibody was stocked in our laboratory. Antibodies against Flag (20543-1-AP), LMAN1 (20543-1-AP), RIG-1/DDX58 (20566-1-AP), IFIH1/MDA5 (21775-1-AP), MAVS (14341-1-AP), IRF3 (11312-1-AP), NF-κB p65 (66535-1-Ig), p-NF-κB p65 (Ser468) (82335-1-RR), and CANX (10427-2-AP / 66903-1-Ig) were obtained from Proteintech. Antibody against p-IRF3 (Ser396) (TA2436S) was obtained from Abmart. Antibodies against Flag (HT201) and GFP (HT801) were obtained from Transgen. Antibodies against GAPDH (YM3029) were obtained from Immunoway. Antibody against β-actin (AB0011) was obtained from Abways. Antibodies against double-stranded RNA (J2) (10010200) were obtained from Scicons. HRP-conjugated secondary antibodies (RS0001/RS0002) were obtained from Immunoway. YSFluor 488 F(ab′)2 Fragment Goat Anti-Mouse (33216ES60)/Rabbit (33116ES60) IgG (H+L), YSFluor 594 Goat Anti-Mouse (33212ES60) IgG (H+L), and YSFluor 647 Goat Anti-Mouse (33213ES60) IgG (H+L) were obtained from Yeasen. Alexa Fluor 568-conjugated anti-rabbit IgG (A-11011) was obtained from Invitrogen. The antibodies were obtained from commercial sources and used following the manufacturer’s instructions.

jetPRIME transfection reagent (101000001) was purchased from Sartorius. Cell lysis buffer (P0013) for western and immunoprecipitant (IP) analyses and phenylmethanesulfonyl fluoride (ST506) were purchased from Beyotime. Phosphatase inhibitor (BL615A), skim milk powder (BS102), and ECL chemiluminescent substrate (BL520B) were purchased from Biosharp. ProteinSafe Protease Inhibitor Cocktail (DI111-01) was purchased from Transgen. RIPA lysis buffer (DXS1515) was purchased from DONGXISW. Omni-Easy Protein Sample Loading Buffer (LT101) was purchased from Epizyme. Protein Marker was purchased from Yeasen (20350ES90) and Vazyme (MP201-01). Stripping buffer (CW0056M) was purchased from CWBIO. Immobilon-PSQ PVDF membranes (ISEQ00010) were purchased from Merck Millipore. Chemical reagents were commercially obtained and used as received.

### Plasmid construction and siRNA synthesis

The open reading frame of *LMAN1* was identified based on the predicted sequence of *Sus scrofa LMAN1* mRNA (GenBank Accession XM_021099870.1). The gene cloning and sequence analysis refer to the previous description. GFP- and Flag-tagged LMAN1 were constructed by cloning the LMAN1 ORF into the pEGFP-N1 and pcDNA3.1(-)-Flag vectors, respectively. The FL, ΔCRD, ΔHelix, N164A, and Vector plasmids were generated by cloning the corresponding swine LMAN1 mutant genes into the pcDNA3.1(+) vector. Plasmids containing point mutations or truncations were generated by overlapping PCR or chemical synthesis. Flag-tagged CSFV proteins (N^pro^, C, E^rns^, E1, E2, p7, NS2, NS3, NS4A, NS4B, NS5A, and NS5B) and GFP-tagged NS5B were constructed and stored in our laboratory.

The shRNA against swine LMAN1 or CMV-FL/CMV-ΔVector were subcloned into the pGreenPuro shRNA (SBI, USA) or pCDH-CMV-MCS-EF1 (SBI, USA) bearing a Green-Puromycin selection marker. Single guide RNAs targeting swine LMAN1 were designed using the CRISPRdirect tool (http://crispr.dbcls.jp/) (20) and cloned into the pLentiCRISPR V2 plasmid encoding the Cas9 bearing puromycin selection markers. Rescue-FL/Rescue-ΔCRD were subcloned into the PiggyBac transposon vector bearing blasticidin S selection markers.

Other plasmids, including pGBPm, pCMV-RIG-I-3×Myc-Puro, pIFNβ-luc, and hRluc/TK were kept in the laboratory. The identity and integrity of each construct were verified by DNA sequencing. All primers used in this study are listed in Table S1.

siRNAs targeting LMAN1 (XM_021099870.1) and MAVS (AB287431.1) were designed using the BLOCK-iT RNAi Designer (Thermo Fisher Scientific, USA). The siRNAs were synthesized by Tsingke, and their sequences are listed in Table S1.

### Cell culture and generation of cell lines

HEK-293T (ATCC; CRL-3216) and porcine kidney (PK-15) (ATCC, CCL-33) cells were cultured in DMEM (Yeasen, 41401ES76) containing 10% fetal bovine serum (FBS) (Zeta-life, Z7186FBS-500/100), 1× Penicillin-Streptomycin (Yeasen, 60162ES76), and 2 mM L-glutamine (Yeasen, 60314ES60). All cells were cultured at 37°C in a 5% CO_2_ incubator.

The lentiviral packaging plasmid(s) constructs were purchased from System Biosciences (SBI, USA). For the generation of PK-15 cells with knockdown or overexpression of LMAN1, lentiviral constructs were co-transfected with packaging plasmids into HEK-293T cells for viral particle production. PK-15 cells were transduced with the harvested particles for 48 h and selected using puromycin.

For the generation of CRISPR-Cas9 LMAN1 knockout cell lines, PK-15 cells infected by lentiviral particles were treated with puromycin (Biosharp, BS111) to select for transduced cells. After screening and cultivation, colonies of surviving cells were digested and inoculated into 96-well plates for single-colony screening and validation.

For the generation of the LMAN1 knockout complementation cell line, PB Transposon Rescue-FL or Rescue-ΔCRD vectors were co-transfected with PiggyBac Transposase vector into LMAN1-KO cells. After 48 h post-transfection, cells were treated with blasticidin S (Beyotime, ST018) to select for transfected cells.

All generated stable cell lines were expanded and frozen for storage, and the efficiency of gene and protein expression in stable cells was tested by RT-qPCR, western blotting, and indirect immunofluorescence. Cell viability was measured by Cell Counting Kit-8 (CCK-8; Biosharp, BS350B) as previously described (21).

### Virus, virus titration, and virus infections

The CSFV Shimen strain (GenBank: AF092448) was grown in PK-15 cells and stored in our laboratory. Virus titers of each treatment were obtained by TCID_50_ assays using the Reed-Muench method, as described previously (21).

### RNA extraction and quantitative real-time PCR (RT-qPCR)

Total RNAs from the samples were extracted using RNA Isolater Total RNA Extraction Reagent (Vazyme, R401), and then reverse-transcribed into cDNA using HiScript II Q RT SuperMix for qPCR (+gDNA wiper) (Vazyme, R223). The qPCR was performed with ChamQ Blue Universal SYBR qPCR Master Mix (Vazyme, Q312) on a CFX96 Touch Real-Time PCR System (Bio-Rad, USA), following the manufacturer’s instructions. Transcription levels were calculated using the 2^−ΔΔct^ method (22), and data were presented as means ± standard deviation (SD). The primers used for qPCR are listed in Table S1.

### RNA sequencing and bioinformatic analysis

Total RNA was extracted from each sample using TRIzol reagent (Invitrogen, 15596018CN) following the manufacturer’s instructions. RNA concentration and purity were measured with a NanoDrop 2000 spectrophotometer (Thermo Fisher Scientific), and RNA integrity was verified on a LabChip GX system (PerkinElmer). RNA-seq libraries were constructed from high-quality RNA with unique index barcodes assigned to each sample. Library quality was evaluated using an Agilent 2100 Bioanalyzer, and qualified libraries were sequenced on an Illumina NovaSeq platform (150-bp paired-end mode) at Biomarker Technologies (Beijing, China).

Raw sequencing reads were processed on the BMKCloud analysis platform (http://www.biocloud.net). Adapter sequences and low-quality reads were removed. Clean reads were aligned to the reference genome using a splice-aware aligner. Gene-level read counts were obtained with featureCounts, and transcript abundance was normalized as fragments per kilobase per million mapped reads. Differentially expressed genes (DEGs) were identified using DESeq2 with thresholds of |log₂ fold change| ≥ 1.5 and adjusted *P* < 0.05.

Functional interpretation of differentially expressed genes was performed using Gene Ontology (GO) classification and Kyoto Encyclopedia of Genes and Genomes (KEGG) pathway enrichment modules implemented on the BMKCloud platform. Volcano plots, heatmaps, and enrichment bar plots were generated using the visualization tools supplied by the same platform, and figures were exported at high resolution for downstream analysis.

### Synchronized CSFV attachment, internalization, and early vRNA replication assay

To synchronize virus entry, cells were pre-chilled at 4°C for 1 h. Free CSFV (multiplicity of infection [MOI] = 20) was diluted in cold infection medium and added to cells. Virus adsorption was carried out for 1 h at 4°C (to allow binding but prevent endocytosis), with gentle rocking to ensure even exposure. After adsorption, cells were washed three times with ice-cold PBS (4°C) to remove unbound virions. Cells collected immediately after the PBS washes were defined as the attachment (0 h) time point. For internalization, the inoculum was replaced with pre-warmed culture medium, and cells were shifted to 37°C to initiate uptake. After 2 h at 37°C, cells were washed with PBS and harvested as the internalization (2 h) time point. To assess early viral RNA replication, infected cells were further incubated at 37°C and harvested at 6 h as the vRNA replication (6 h) time point. At each time point, CSFV vRNA levels were quantified by RT-qPCR.

### Western blot

Protein samples were obtained by lysing the cells on ice, and then mixed with 5× SDS loading buffer (Epizyme, LT101) and boiled. Proteins were separated by electrophoresis using SDS-PAGE, then transferred onto PVDF membranes. Following transfer, the membranes were blocked with 5% skim milk at room temperature for 2 h. Primary antibodies were incubated overnight at 4°C, and secondary antibodies were incubated for 1 h at room temperature. Antibodies were diluted in TBST, and each step was followed by three washes with TBST. Membranes were developed using chemiluminescence and visualized by Amersham ImageQuant 800 (Cytiva, USA) according to the manufacturer’s instructions. Signals were quantified by ImageJ (National Institutes of Health, USA) with the gel tool.

### Immunoprecipitations

HEK-293T cells were transfected with the indicated plasmids for 48 h. Cells were collected, washed, and treated with lysis buffer on ice for 30 min. The centrifugal collection of supernatants was incubated with anti-Flag antibody for 1 h at 4°C. Subsequently, the mixture was incubated with Protein A/G PLUS-Agarose (Santa, sc-2003) overnight at 4°C. The immunoprecipitates were washed with PBS and collected by centrifugation, and then resuspended in 1× SDS loading buffer and boiled for 10 min. Finally, the proteins were analyzed by SDS-PAGE and Western blotting.

### Immunofluorescence assay

For immunofluorescence, cells were seeded on coverslips and transfected if necessary, then washed with PBS and fixed with 4% paraformaldehyde (Biosharp, BL539A), permeabilized in PBS with 0.3% Triton X-100 (Solarbio, T8200). After being blocked with 3% bovine serum albumin (MPBIO, 02FC0077) for 2 h at room temperature, the samples were incubated with diluted primary antibodies overnight at 4°C, and fluorescent secondary antibodies were incubated 1 h at room temperature. Finally, samples were stained with 4′,6-diamidino-2-phenylindole hydrochloride (DAPI) (Beyotime, C1005), and then mounted onto glass slides using antifade solution (Solarbio, S2100). Cell slides were washed three times with PBST between steps. Fluorescence signals were visualized with an epifluorescence microscope (Zeiss Observer 3, Germany) or a confocal microscope (Leica TCS SP8, Germany).

### Immunohistochemistry

Fresh tissue samples from the specified organs were collected from treated animals and fixed in 4% paraformaldehyde. The fixed tissues were washed with running water overnight, followed by dehydration through a graded ethanol series. The tissues were then clarified with fresh xylene and embedded in paraffin blocks. Paraffin-embedded tissue samples were sectioned at 4 μm thickness, and immunostained using a rabbit anti-LMAN1 monoclonal antibody after deparaffinization and antigen unmasking. The immunohistochemistry kit (Servicebio, G1215) was utilized for staining. After counterstaining with hematoxylin for 3 min, sections were dehydrated and examined under a Nikon Eclipse Ni-U biological microscope (Japan) at a magnification of 200×. The appreciable brown staining was considered as detection of LMAN1 protein.

### Crude replication complex (CRC) extraction

CSFV CRC was extracted by reference to the previous method (6). Briefly, cells were infected with CSFV (MOI of 1 and 10), or left uninfected as controls. After 24 h, cells were harvested and washed with pre-cooled PBS. The collected cells were centrifuged at 800 × *g* for 10 min, and the pellet was resuspended in hypotonic buffer (Biorigin, BN24371) to induce swelling. The suspension was then homogenized and subjected to centrifugation at 1,000 × *g* for 10 min. The supernatant was subsequently centrifuged at 68,500 × *g* for 1 h to isolate the CRC (23, 24). The final CRC pellet was resuspended in 1× SDS loading buffer and stored at −80°C for further analysis. Finally, LMAN1, calnexin, and β-actin were detected by western blot.

### IFN-β dual-luciferase assay

The IFN-β promoter activity was evaluated using a dual-luciferase reporter system. Briefly, PK-15 cells were co-transfected with a firefly luciferase reporter plasmid containing the IFN-β promoter (pIFNβ-Luc) and a Renilla luciferase control plasmid (hRluc-TK), then stimulated with CSFV (MOI = 10) or poly(I:C) (1 μg/mL). Firefly and Renilla luciferase activities were sequentially measured using a Dual-Luciferase reporter assay kit (Promega, E1910) following the manufacturer’s instructions. Luminescence was read using Spark multi-functional enzyme labeling instrument (Tecan, Germany).

### ELISA

To quantify IFN-β production, supernatants from cells stimulated with CSFV (MOI = 10) or poly(I:C) (1 μg/mL) were collected at 6 h or 12 h, respectively. IFN-β concentrations were measured using a porcine IFN-β ELISA kit (mbbiology, MB-5136A) according to the manufacturer’s instructions. Absorbance was read at 450 nm using a microplate reader (Tecan, Germany).

### Statistical analysis

All data are presented as mean ± SD derived from three independent experiments. Significant differences were tested with a one-way ANOVA followed by *post hoc* Tukey tests and two-tailed *t*-tests (*P* < 0.05). The *P*-values were as follows: 0.01 ≤ *P < 0.05, **P < 0.01 and no significant difference (ns) means *P* > 0.05.

## RESULTS

### Expression patterns of LMAN1 after CSFV infection

**Fig 1:**
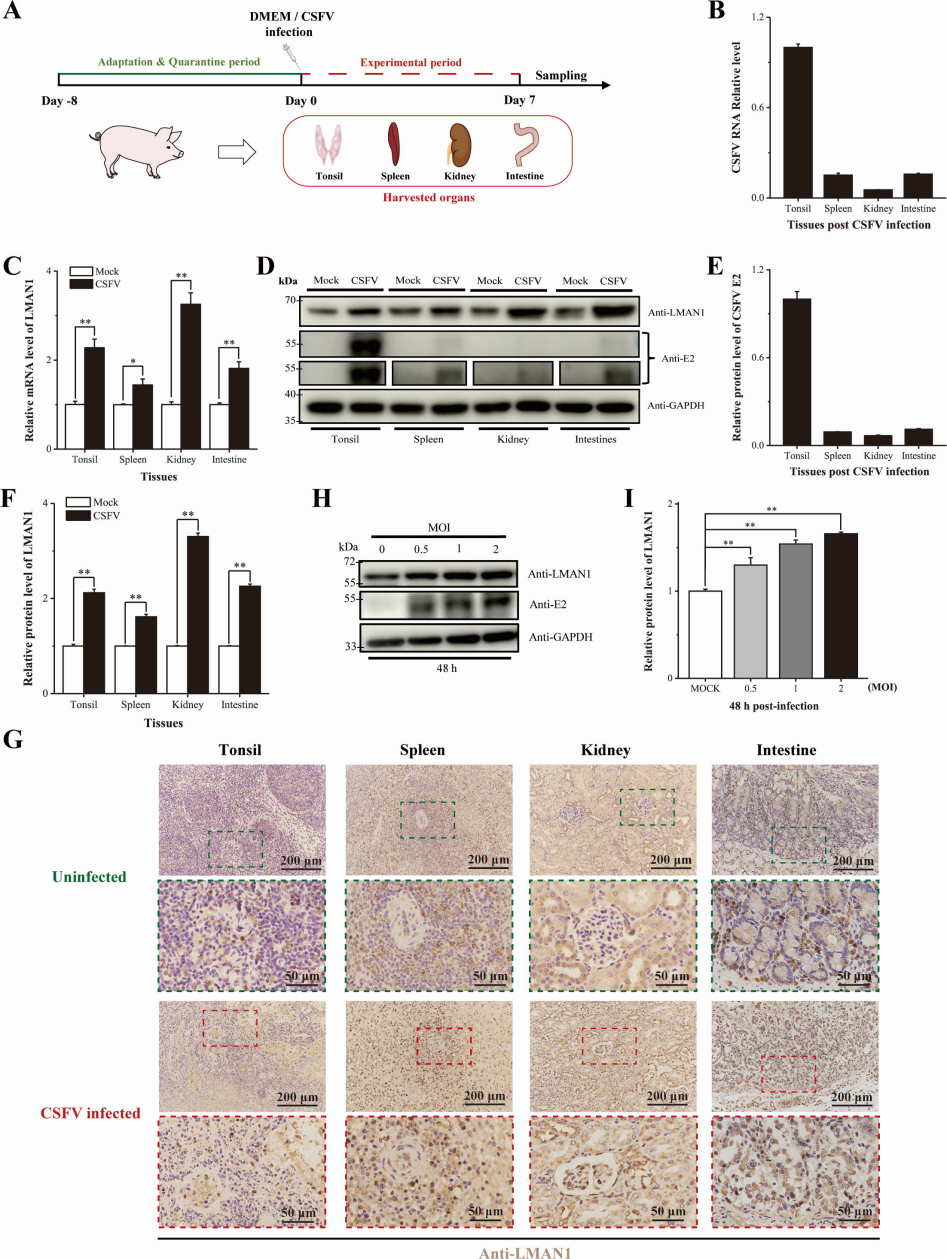
CSFV infection increases the expression of LMAN1 *in vivo* and *in vitro*. (**A**) Schematic overview of the piglet infection experiment and organ collection. (**B and C**) The mRNA levels of NS5B and LMAN1 in tissues assessed by RT-qPCR. (**D–F**) Detection and assessment of LMAN1 levels after CSFV infection by western blot. The band intensities were semi-quantified using ImageJ. (**G**) Expression of LMAN1 detected in harvested organs by immunohistochemistry. (**H and I**) Detection and measurement of LMAN1 protein levels in PK-15 cells infected with CSFV by western blot. The band intensities were semi-quantified using ImageJ. Error bars represent standard deviation (*n* = 3). No significance (ns) means *P* > 0.05; * means 0.01 ≤ *P* < 0.05; ** means *P* < 0.01 (two-way ANOVA).

In order to examine the LMAN1 expression upon CSFV infection, an animal infection model was firstly established. Six piglets were randomly divided into two groups, challenged with either CSFV or DMEM (as the control), and the body temperature of the experimental pigs was monitored for 7 days. Then the piglets were euthanized and tissue samples were collected (Fig. 1A). During the infection phase, the control group maintained a normal and stable rectal temperature, whereas the CSFV-infected group developed fever, with the mean body temperature peaking at 41.3°C on 4 dpi and remaining between 40.3 and 41.3°C thereafter, indicating persistent high pyrexia (Fig. S1A). During the observation period, the CSFV-infected group displayed clinical signs and gross lesions typical of CSFV, while no obvious abnormalities were observed in the control group. Therefore, we successfully established a model of CSFV infection in pigs. And then, the expression of the CSFV E2 gene in the sampled tissues (tonsils, spleen, kidney, and intestine) was confirmed via PCR testing, further validating the infection status of CSFV in pigs (Fig. S1B).

The expression patterns of LMAN1 in these tissues were measured via RT-qPCR and western blot. The detection of CSFV genomic RNA (represented by the NS5B fragment) by RT-qPCR and the detection of viral protein (the E2 protein) expression by western blot confirmed that the viral content was the highest in tonsils (Fig. 1B, D, and E). Additionally, host LMAN1 was upregulated in all examined tissues following CSFV infection, and the expression level of LMAN1 was found to be the highest in kidney (Fig. 1C, D, and F). Furthermore, changes in LMAN1 protein expression pattern in these tissues were detected by IHC, which showed that the distinct dark brown-labeled LMAN1 signal was upregulated in the sampled tissues upon CSFV infection in comparison to the control group (Fig. 1G). Finally, we also infected an immortalized PK-15 (Pig Kidney-15) cell line with CSFV at different multiplicities of infection (MOI) to determine the expression of LMAN1. Inconsistent with the animal infection data, the protein levels of LMAN1 increased after CSFV infection at all the checked time points (Fig. 1H and I; Fig. S1C and D). Taken together, our animal and cell infection models showed that CSFV infection enhances expression of host LMAN1.

### LMAN1 facilitates CSFV proliferation at vRNA replication stage

The upregulation of LMAN1 expression upon CSFV infection suggests its potential involvement in CSFV proliferation. To investigate this, we reduced the LMAN1 levels by synthetic siRNA-mediated knockdown (Fig. S2) or enhanced cellular LMAN1 levels by transient overexpression in PK-15 cells. The preliminary results suggested that LMAN1 promotes the infection of CSFV (Fig. S3). To achieve a homogeneous reduction in LMAN1 levels across the entire cell population, we designed an shRNA targeting LMAN1 mRNA (shLMAN1) and generated PK-15 cells stably expressing either shLMAN1 or shN (a non-targeting control shRNA). Cell clones were screened and characterized, showing unchanged cell viability and stable reduction of LMAN1 on both mRNA and protein levels (Fig. S4A through D). In parallel, cells stably overexpressing LMAN1 (Flag-LMAN1) were generated, which also showed significant overexpression in comparison to control while the cell viability was not impaired (Fig. S4E through I).

**Fig 2:**
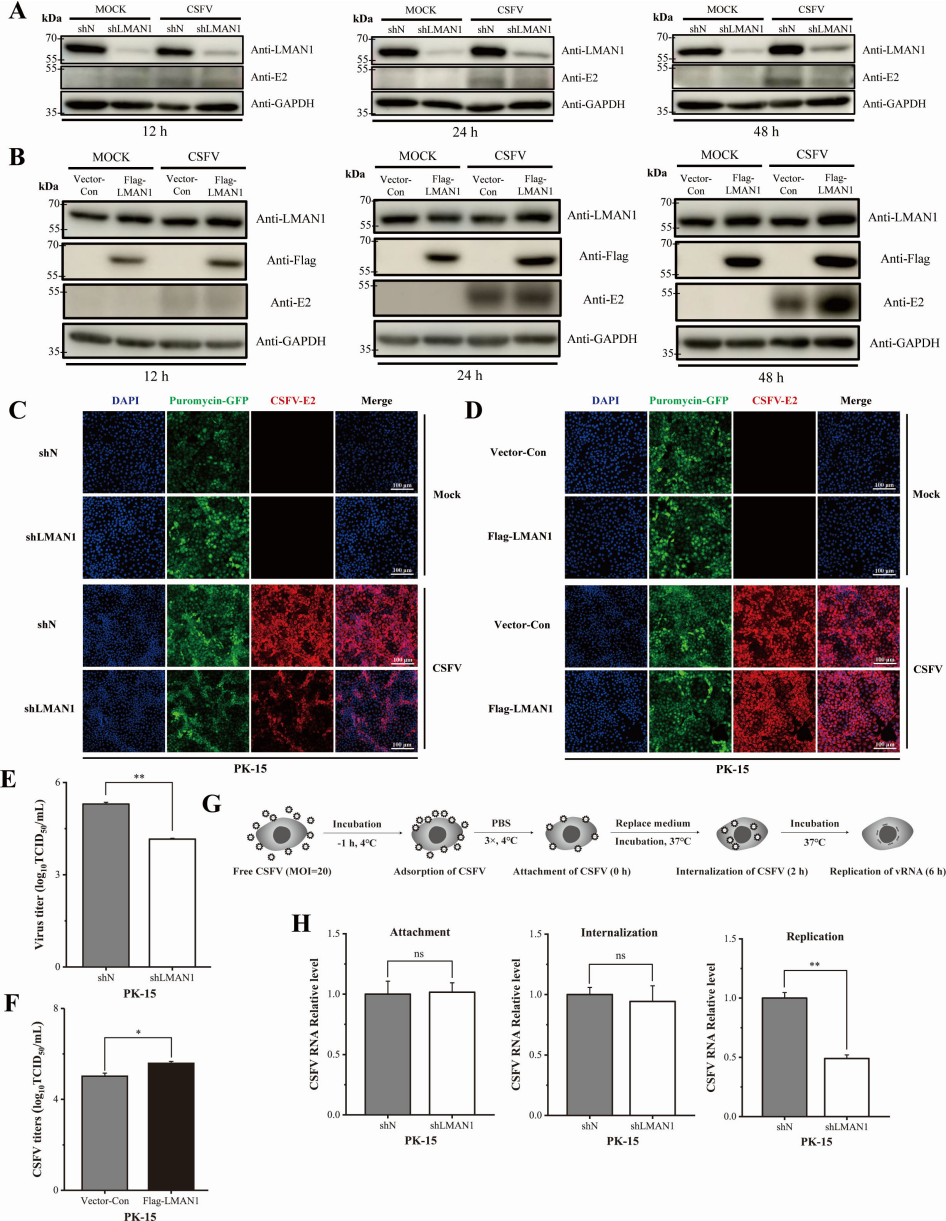
LMAN1 expression is required for efficient CSFV infection. (**A and B**) Viral E2 protein immunoblotted in PK-15 cells stably knocking down or overexpressing LMAN1 after CSFV infection (MOI = 1) for 12, 24, and 48 h. shLMAN1, cells stably expressing shRNA targeting LMAN1; shN, nonspecific shRNA control; Flag-LMAN1, Flag-tagged LMAN1; Vector-Con, empty vector control. (**C and D**) Immunofluorescence detection of E2 protein in PK-15 cells stably knocking down or overexpressing LMAN1 after CSFV infection (MOI = 10) for 24 h. Puromycin-GFP serves as a marker for ectopic shRNA or protein expression. Scale bars = 100 µm. (**E and F**) Titers of CSFV progeny virus in PK-15 cells with LMAN1 knockdown or overexpression. Viral titers were determined by endpoint dilution based on immunofluorescence detection. (**G**) Schematic diagram of cell infection and sample collection. (**H**) Virus attachment, internalization, and vRNA replication measured after LMAN1 knockdown. Error bars represent standard deviation (*n* = 3). No significance (ns) means *P* > 0.05; * means 0.01 ≤ *P* < 0.05; ** means *P* < 0.01.

Next, CSFV was inoculated into shLMAN1 or Flag-LMAN1 cells, and E2 protein was detected at various time points as an indication of viral proliferation. While the reduction of LMAN1 protein led to less viral E2 protein amount than in the shN control (Fig. 2A), higher E2 protein level was observed in PK-15 cells overexpressing LMAN1 than in control cells at all three checked time points (Fig. 2B). Immunofluorescence detection of viral E2 protein in the infected cells also supported that LMAN1 promotes viral proliferation (Fig. 2C and D). Furthermore, production of progeny virus was tested via determination of its TCID_50_, which showed that knockdown and overexpressed LMAN1 elevated and reduced production of CSFV progeny virus, respectively (Fig. 2E and F). Our transient and stable knockdown/overexpression experiments demonstrated that LMAN1 is required for efficient CSFV proliferation, confirming its important role in viral infection.

To further characterize the function of LMAN1 on CSFV infection, we checked the important stages of CSFV infection, including virus attachment, internalization, and viral RNA (vRNA) replication by quantification of the genomic RNA (Fig. 2G and H). While attachment and internalization of the CSFV virion were not changed by reduction of LMAN1, genomic RNA replication was significantly impaired (Fig. 2H). Collectively, our data demonstrated that LMAN1 supports viral genomic RNA replication and facilitates CSFV proliferation.

### LMAN1 interacts and colocalizes with CSFV RNA polymerase NS5B

**Fig 3:**
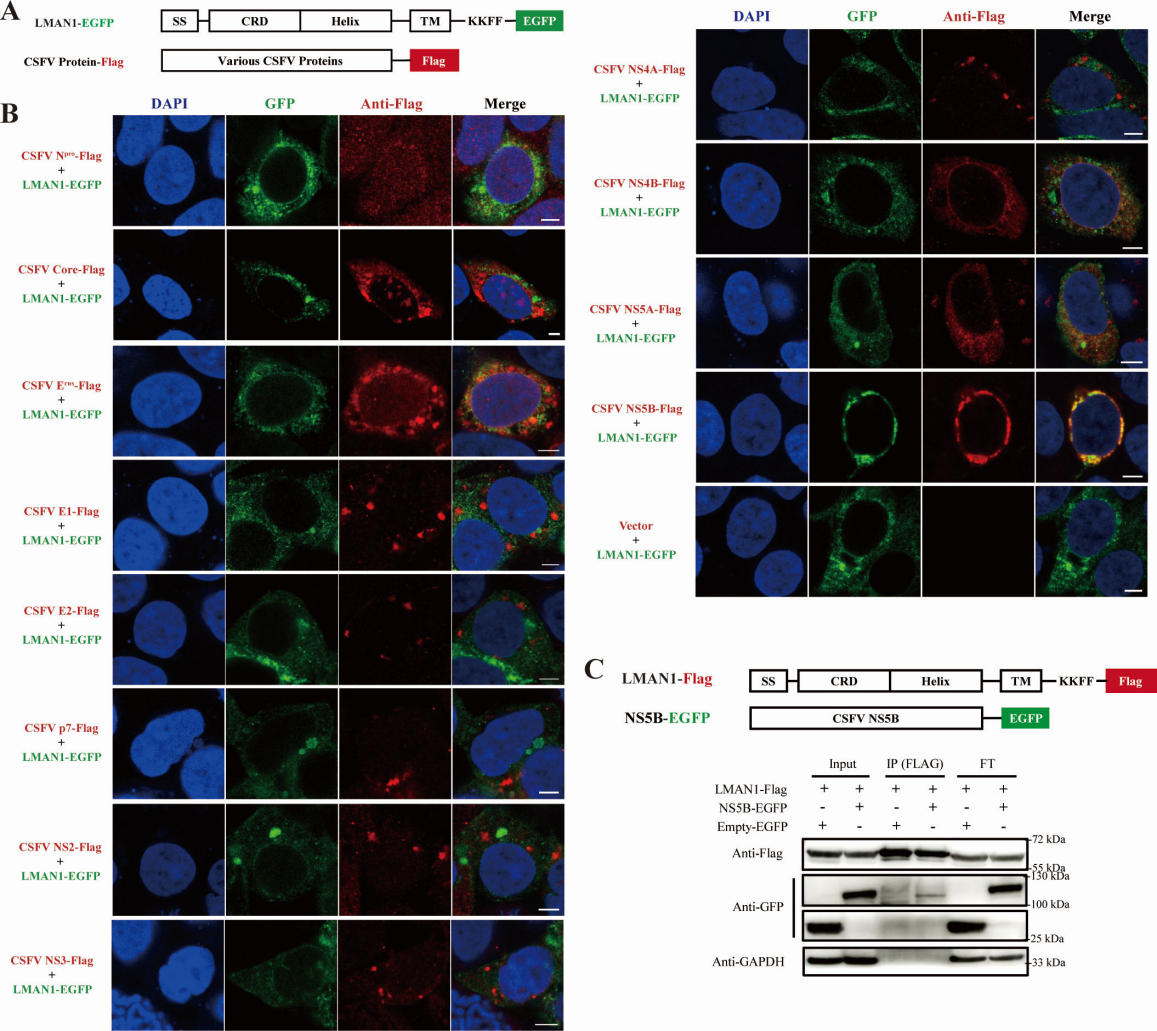
Interaction analysis of LMAN1 with CSFV proteins. (**A**) Schematic representation of LMAN1-GFP and CSFV protein constructs used for co-localization assays. SS, signal sequence; CRD, carbohydrate recognition domain; Helix, helical or stalk domain; TM, transmembrane; KKFF, KKFF motif. (**B**) Representative images showing cellular distribution and localization of LMAN1 and individual CSFV proteins. LMAN1-GFP and CSFV proteins (N^pro^, Core, E^rns^, E1, E2, p7, NS2, NS3, NS4A, NS4B, NS5A, NS5B) were co-expressed in cells, and Flag-tagged viral proteins were visualized by immunofluorescence staining using a mouse anti-Flag antibody. Scale bars = 10 µm. (**C**) Interactions between NS5B and LMAN1 examined by co-immunoprecipitation. Whole-cell lysates (Input) and anti-Flag IP or flow-through (FT) were probed with anti-GFP, anti-Flag, or anti-GAPDH antibodies as indicated.

To better understand the role that LMAN1 plays in CSFV replication process, we examined the co-localization of LMAN1 and CSFV proteins using laser confocal microscopy. Plasmids encoding LMAN1-EGFP, together with each of the Flag-tagged 12 viral proteins of CSFV, including both structural (Core/E^rns^/E1/E2/p7) and non-structural (N^pro^/NS2/NS3/NS4A/NS4B/NS5A/NS5B) proteins (Fig. 3A), were co-transfected into HEK-293T cells and subjected to confocal microscopy for their localization. The viral proteins showed different cellular distribution, mainly in the cytoplasm, and the NS5B protein modulated cellular distribution of LMAN1-GFP and showed strong co-localization with LMAN1 (Fig. 3B), indicating a potential interaction between the two proteins. Co-immunoprecipitation experiments were performed with lysate from LMAN1-Flag and NS5B-EGFP co-transfected HEK-293T cells, which showed that NS5B-GFP was co-precipitated with LMAN1, confirming the interaction between LMAN1 and NS5B (Fig. 3C).

### LMAN1 binds to CSFV NS5B in a CRD-dependent manner

**Fig 4:**
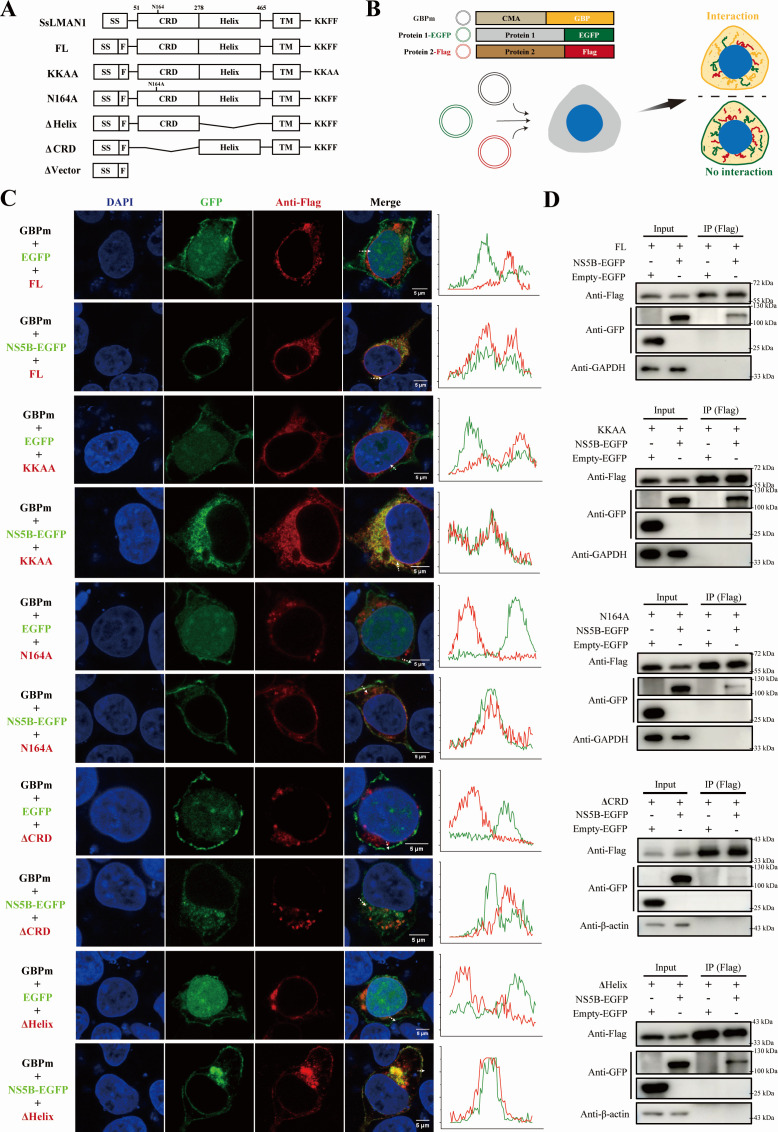
LMAN1 interacts with NS5B in a CRD-dependent manner. (**A**) Schematic diagram of SsLMAN1 and the corresponding mutants constructed for protein interaction assays. SS, signal sequence; F, Flag epitope tag; CRD, carbohydrate recognition domain; Helix, helical or stalk domain; TM, transmembrane; KKFF motif. (**B**) Schematic representation of the translocation PPI assay. The membrane-anchored GFP-binding protein (mGBP), composed of a cell-membrane-anchoring domain (CMA) and a GFP-binding nanobody (GFP binding protein, GBP), re-localizes GFP-tagged proteins to the plasma membrane. Flag-tagged proteins can be recruited and colocalized with their GFP-tagged interactors on cell membrane (upper cell, indication of interaction), or dispersed without colocalization (lower cell, no interaction). (**C**) Interactions between CSFV NS5B and LMAN1 mutants visualized by the translocation PPI assay. Representative images of NS5B and LMAN1 mutants co-localization on cell membranes are shown, and a line profile of fluorescence intensity across the cell membrane was plotted. The white arrow indicates the analyzed area. Scale bars = 5 µm. (**D**) Validation of the interaction between NS5B and LMAN1 mutants by Co-IP. LMAN1 mutants were Flag-tagged, and co-precipitated NS5B was blotted via fused GFP (anti-GFP).

To determine the molecular basis for LMAN1’s association with CSFV NS5B, we constructed a panel of LMAN1 truncates and mutants based on protein sequence analyses. Alignment of SsLMAN1 (*Sus scrofa* LMAN1) and HsLMAN1 (*Homo sapiens*) protein sequences showed 91.89% similarity between the two proteins (Fig. S5A), and the SsLMAN1 (518 amino acid residues in total), contains an N-terminal signal sequence (SS) (residue 1–30), a CRD (residue 51–278), a helical or stalk domain (Helix) (residue 278–465), a transmembrane region (TM) (residue 484–506) and a KKFF sequence, suggested by its similarity to the human ortholog. Therefore, truncation mutants lacking the CRD or Helix domain, as well as point mutants with impaired ERGIC localization (KKAA) or sugar-binding capacity (N164A), were constructed, and their expression was verified by Western blotting (Fig. 4A; Fig. S5B through D). When co-transfected, deletion of the CRD domain (ΔCRD) led to loss of its colocalization with NS5B, but not other mutants, demonstrating the important role of the CRD domain in their interaction (Fig. S6).

**Fig 5:**
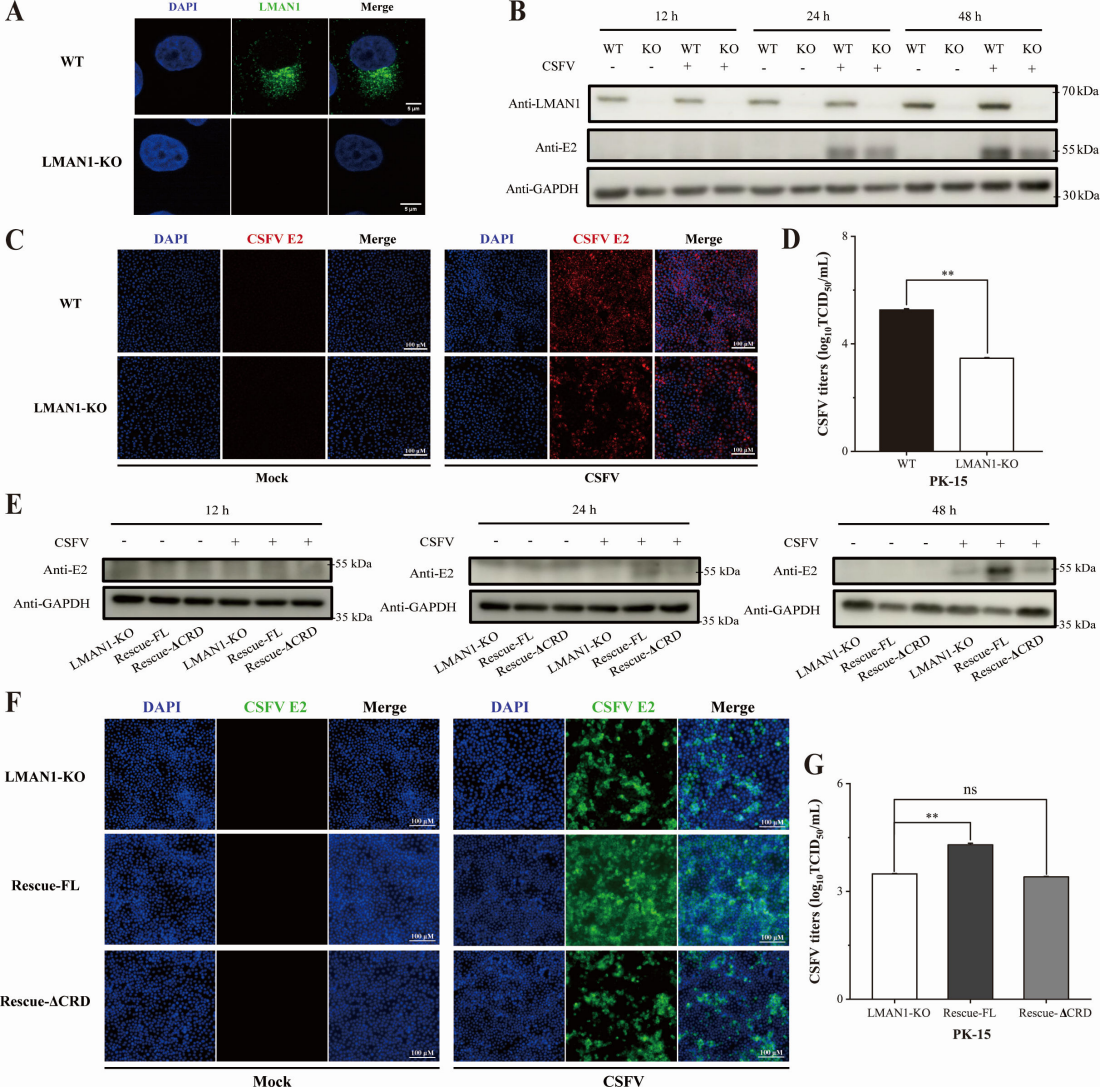
LMAN1 regulates CSFV replication through its CRD. (**A**) Identification of LMAN1 knockout cells by immunostaining. (**B**) CSFV proliferation in LMAN1-KO cells. The E2 protein of CSFV was detected as an indication of virus propagation. (**C**) CSFV propagation in LMAN1-KO cells detected by immunofluorescence. Viral E2 protein was detected with a mouse anti-CSFV E2 antibody, and nuclei were counterstained with DAPI. Scale bars = 100 µm. (**D**) Viral titers in WT and LMAN1-KO cells infected with CSFV for 24 h. (**E**) Proliferation of CSFV in cells reintroduced with full-length (Rescue-FL) or ΔCRD (Rescue-ΔCRD) LMAN1. (**F**) CSFV propagation in LMAN1-KO, Rescue-FL, and Rescue-ΔCRD cells detected by immunofluorescence. Scale bars = 100 µm. (**G**) Viral titers in LMAN1-KO, Rescue-FL, and Rescue-ΔCRD cells infected with CSFV for 24 h, determined using immunofluorescence. Error bars represent standard deviation (*n* = 3). No significance (ns) means *P* > 0.05; * means 0.01 ≤ *P* < 0.05; ** means *P* < 0.01.

Next, interaction between the truncates or mutants and NS5B was detected with a translocation-based protein-protein interaction (PPI) assay. In this assay, plasmids encoding NS5B, LMAN1 mutants, as well as a membrane-bound GFP binding nanobody (membrane GFP binding protein, mGBP) were triply transfected into cells for protein expression (Fig. 4B). The expressed NS5B-GFP protein was anchored onto the inner side of the cell membrane by the mGBP. LMAN1 mutants, which physically interact with NS5B, could be recruited to the membrane via the interaction, thus displaying colocalization on the cell membrane, indicating an interaction between the two proteins (Fig. 4B). As shown in Fig. 4C, all LMAN1 constructs except for the ΔCRD were recruited and colocalized with NS5B at the cell membranes, revealing the essential role of the CRD domain for NS5B binding. Meanwhile, we also detected abolished interaction by depletion of CRD with co-immunoprecipitation (Fig. 4D), further confirming our findings. Taken together, our results demonstrated that the CRD of LMAN1 was indeed essential for binding to CSFV NS5B.

### LMAN1 affects the proliferation of CSFV through its CRD domain

To test whether the CRD domain of LMAN1 is required for CSFV infection, we used CRISPR-Cas9 gene editing technology to generate PK-15 cells lacking LMAN1. LMAN1 knockout cells (LMAN1-KO) were identified by genome sequencing and further characterized for their viability and LMAN1 deficiency using immunoblotting and immunofluorescence. These analyses confirmed homozygous editing and complete loss of LMAN1 expression in the LMAN1-KO cells (Fig. 5A; and Fig. S7A through D). We then infected LMAN1-KO or unedited control (wild-type, WT) cells and monitored the virus proliferation by immunoblotting (Fig. 5B) and immunofluorescence (Fig. 5C). Loss of LMAN1 significantly reduced CSFV proliferation, and accordingly, progeny virus production was impaired (Fig. 5D). We next reintroduced LMAN1 full-length (FL) or ΔCRD protein into the knockout cells. Cells rescued with FL or ΔCRD LAMN1 showed good viability, stably expressing the introduced protein (Fig. S7E through H). As expected, while full-length LMAN1 restored CSFV infection, the ΔCRD did not reverse the reduction of viral infection resulting from LMAN1 knockout (Fig. 5E through G). Our data demonstrated that the CRD domain of LMAN1, which is responsible for interacting with CSFV NS5B, is also indispensable for facilitating CSFV replication.

### LMAN1 is recruited to the CSFV replication complex and facilitates efficient viral RNA synthesis

**Fig 6:**
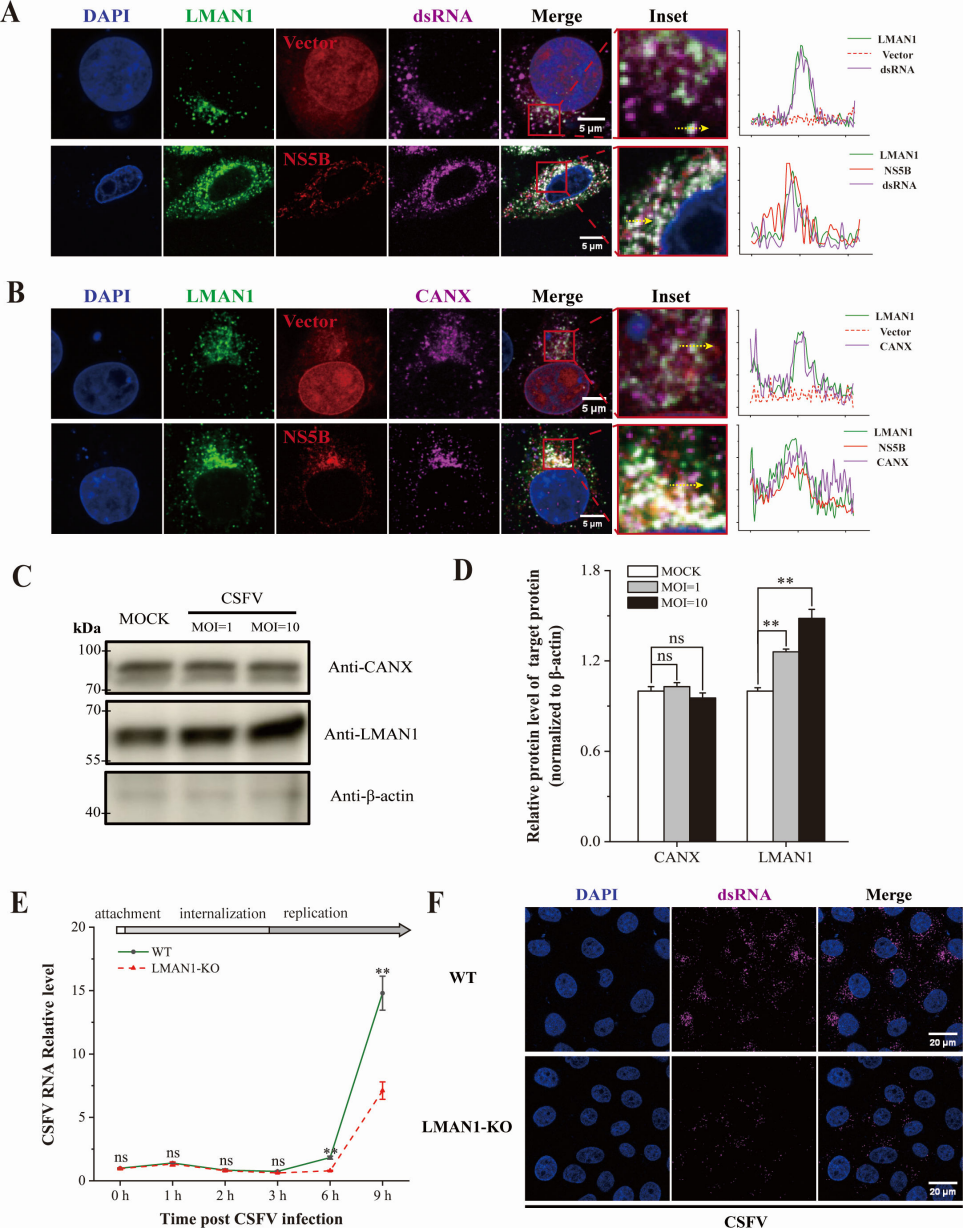
LMAN1 participates in the formation of the CSFV replication complex. (**A and B**) Colocalization of LMAN1 and virus replication sites. As the replicative intermediates, dsRNA was detected as a marker for vRNA replicating sites (**A**), and CANX protein was stained for the ER where CSFV replication occurs (**B**). Line profile of fluorescence intensity across the cell membrane was plotted, and the yellow arrow indicates the analyzed area. Scale bars = 5 µm. (**C and D**) Detection of LMAN1 in crude replication complex of CSFV. Higher LMAN1 amount was detected in crude replication complex in cells inoculated at an MOI of 10 than that of 1. (**E**) Impaired stages of viral life cycle caused by LMAN1 deficiency. While no significant difference was observed at early stages of virus infection, significantly lower viral RNA was detected in LMAN1-KO than in WT cells after RNA replicating around 6 hpi. (**F**) Reduction of viral genome replication sites in LMAN1-KO cells. dsRNA was produced as intermediates of vRNA replication and detected with a specific antibody to visualize viral replication factories. Scale bars = 20 µm. Error bars represent standard deviation (*n* = 3). No significance (ns) means *P* > 0.05; * means 0.01 ≤ *P* < 0.05; ** means *P* < 0.01.

To determine whether LMAN1 participates in the formation of the CSFV replication complex, we first examined its subcellular localization during infection. In PK-15 cells transfected with NS5B-GFP and infected with CSFV, strong colocalization was observed between LMAN1 and the cytosolic dsRNA signals. Since dsRNA was produced as an intermediate during replication of the single-strand genomic RNA of CSFV, this colocalization suggests LMAN1 may participate in the viral RNA synthesis directly (Fig. 6A). LMAN1 also colocalized with the ER marker CANX within these structures (Fig. 6B), suggesting that LMAN1 is recruited to ER-derived replication membranes. In contrast, pEGFP-N1–transfected control cells showed no such colocalization, indicating that LMAN1 enrichment is specifically associated with NS5B expression and CSFV replication.

We next assessed whether CSFV infection modulates LMAN1 presence within the replication complex. Western blot analysis of isolated replication-complex fractions showed a dose-dependent increase of LMAN1 in response to CSFV infection at MOI = 1 and 10, while the ER marker CANX remained unchanged (Fig. 6C and D).

Quantification of intracellular viral RNA revealed significantly lower vRNA was detected in LMAN1-KO cells than in WT cells after 6 hpi, from which viral RNA replication occurs, indicating loss of LMAN1 impairs viral RNA synthesis but not early stages of infection (Fig. 6E). Consistently, confocal imaging at 12 hpi showed markedly fewer and smaller dsRNA-positive replication factories in KO cells, indicating that LMAN1 contributes to efficient formation or stability of replication organelles (Fig. 6F; Fig. S8).

We further examined whether the viral RNA-dependent RNA polymerase NS5B regulates LMAN1 abundance. NS5B-GFP expression induced a time-dependent upregulation of endogenous LMAN1 protein, whereas cells expressing the GFP vector showed no change (Fig. S9A). A similar effect was observed with NS5B-Flag, but the catalytically inactive NS5B mutant (GDD to GAA) (25), also increased LMAN1 levels (Fig. S9B), demonstrating that the RNA polymerase activity of NS5B is not required for LMAN1 induction.

Collectively, these data demonstrated that LMAN1 is recruited to the membranous CSFV replication sites and enriched in viral replication-associated protein fractions, serving for efficient viral RNA replication.

### LMAN1 deficiency amplifies antiviral transcriptional responses during CSFV infection

**Fig 7:**
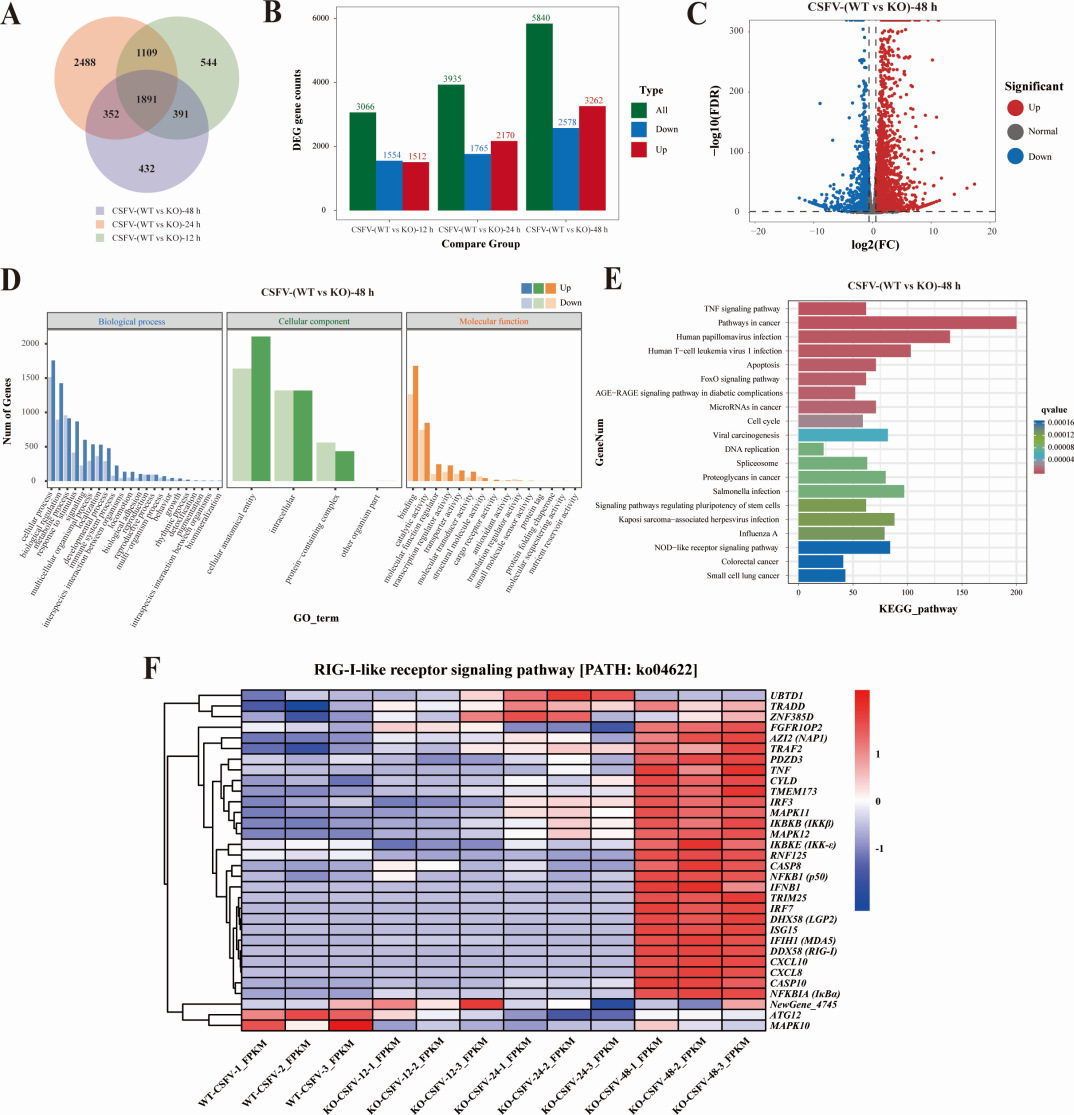
RNA-seq data in LMAN1-deficient cells following CSFV infection. (**A**) Venn diagram showing the overlap of DEGs between CSFV-infected WT and LMAN1-KO cells at 12, 24, and 48 hpi. Each circle represents the set of DEGs identified at the indicated time point, and the numbers denote genes uniquely or commonly shared among the three comparisons. (**B**) DEGs between CSFV-infected WT and LMAN1-KO cells at 12, 24, and 48 hpi. The bar graph shows the total number of DEGs (green) and the numbers of significantly downregulated (blue) and upregulated (red) genes in each comparison group (WT vs KO) at the indicated time points. (**C**) Volcano plot of DEGs between CSFV-infected WT and LMAN1-KO cells at 48 hpi. Red dots indicate significantly upregulated genes, blue dots indicate significantly downregulated genes, and gray dots represent non-significant genes. The vertical and horizontal dashed lines denote the fold change and FDR thresholds used to define differential expression. (**D**) GO enrichment analysis of differentially expressed genes between CSFV-infected WT and LMAN1-KO cells at 48 hpi. Significantly enriched GO terms are shown for the three main categories: Biological process, Cellular Component, and Molecular Function. The intensity of the color represents the upregulation (deep) and downregulation (light) of the genes. (**E**) KEGG pathway enrichment of DEGs between CSFV-infected WT and LMAN1-KO cells at 48 hpi. The horizontal bar plot shows the top 20 significantly enriched KEGG pathways. Bar colors represent the adjusted *q* values, as shown in the scale on the right, with warmer colors indicating higher statistical significance. (**F**) Heatmap of DEGs in the RIG-I-like receptor signaling pathway (ko04622), showing coordinated upregulation of key antiviral sensors, adaptors, kinases, transcription factors, and effector genes in LMAN1-KO cells, suggesting enhanced innate immune activation during CSFV infection. Expression values are displayed as Z-score-scaled TPM values.

To define the impact of LMAN1 on host transcriptional profiles during CSFV infection, RNA-seq was performed in WT and LMAN1-KO cells at 12, 24, and 48 h post-infection (hpi). Viral infection conditions were optimized for RNA-seq (Fig. S10A) and then infected samples were collected and subjected to sequencing and analysis. A Venn diagram analysis revealed both substantial overlap and time-specific transcriptional responses, with 1,891 DEGs shared among all three time points and additional sets of genes uniquely altered at 12, 24, or 48 hpi (Fig. 7A). Consistent with a progressively amplified response, the total number of DEGs increased over time, from 3,066 genes at 12 hpi to 3,935 genes at 24 hpi and 5,840 genes at 48 hpi (Fig. 7B). At each time point, both up- and downregulated genes were detected, with a modest predominance of upregulated genes (1,512 up vs 1,554 down at 12 hpi; 2,170 up vs 1,765 down at 24 hpi; and 3,262 up vs 2,578 down at 48 hpi), indicating that LMAN1 deficiency reshapes the host transcriptional landscape in a dynamic and time-dependent manner during CSFV infection.

Volcano plots further illustrated these transcriptomic changes, showing widespread and progressively pronounced shifts in gene expression between WT and LMAN1-KO cells as infection proceeded (Fig. 7C; Fig. S10B). With the predefined log2 fold-change and FDR thresholds, a large number of genes were significantly upregulated (red) or downregulated (blue) in LMAN1-KO cells, particularly at 48 hpi, whereas non-significant genes (gray) clustered tightly around the origin (Fig. 7C). Functional annotation of DEGs by GO enrichment demonstrated that altered genes were distributed across all three major GO categories—Biological Process, Cellular Component, and Molecular Function—with the number of enriched terms and involved genes increasing from 12 to 48 hpi (Fig. 7D; Fig. S10C). Finally, KEGG pathway enrichment analysis identified the top 20 significantly over-represented pathways at each time point (Fig. 7E; Fig. S10D), including numerous signaling and disease-associated pathways such as cancer- and infection-related pathways, TNF and Wnt signaling, ECM-receptor interaction, apoptosis, cell cycle regulation, and innate immune signaling. Together, these data indicated that loss of LMAN1 profoundly and progressively remodels host gene expression programs and impacts multiple signaling and stress-response pathways during CSFV infection.

KEGG analyses in detail showed DEGs within the RIG-I-like receptor signaling pathway (ko04622) were generally upregulated. Heatmap visualization revealed coordinated upregulation of key antiviral sensors (RIG-I and MDA5), adaptors (MAVS), transcription factors (IRF3), and downstream effectors in KO cells at 48 hpi (Fig. 7F). This pathway-wide enhancement indicates that LMAN1 acts as a negative regulator of antiviral innate immunity and that its deficiency leads to amplified activation of RLR signaling upon CSFV challenge.

Collectively, these transcriptomic findings demonstrate that LMAN1 loss reprograms host antiviral gene expression, resulting in heightened innate immune activation during CSFV infection.

### LMAN1 deficiency potentiates RLR signaling and strengthens antiviral responses during CSFV infection

**Fig 8:**
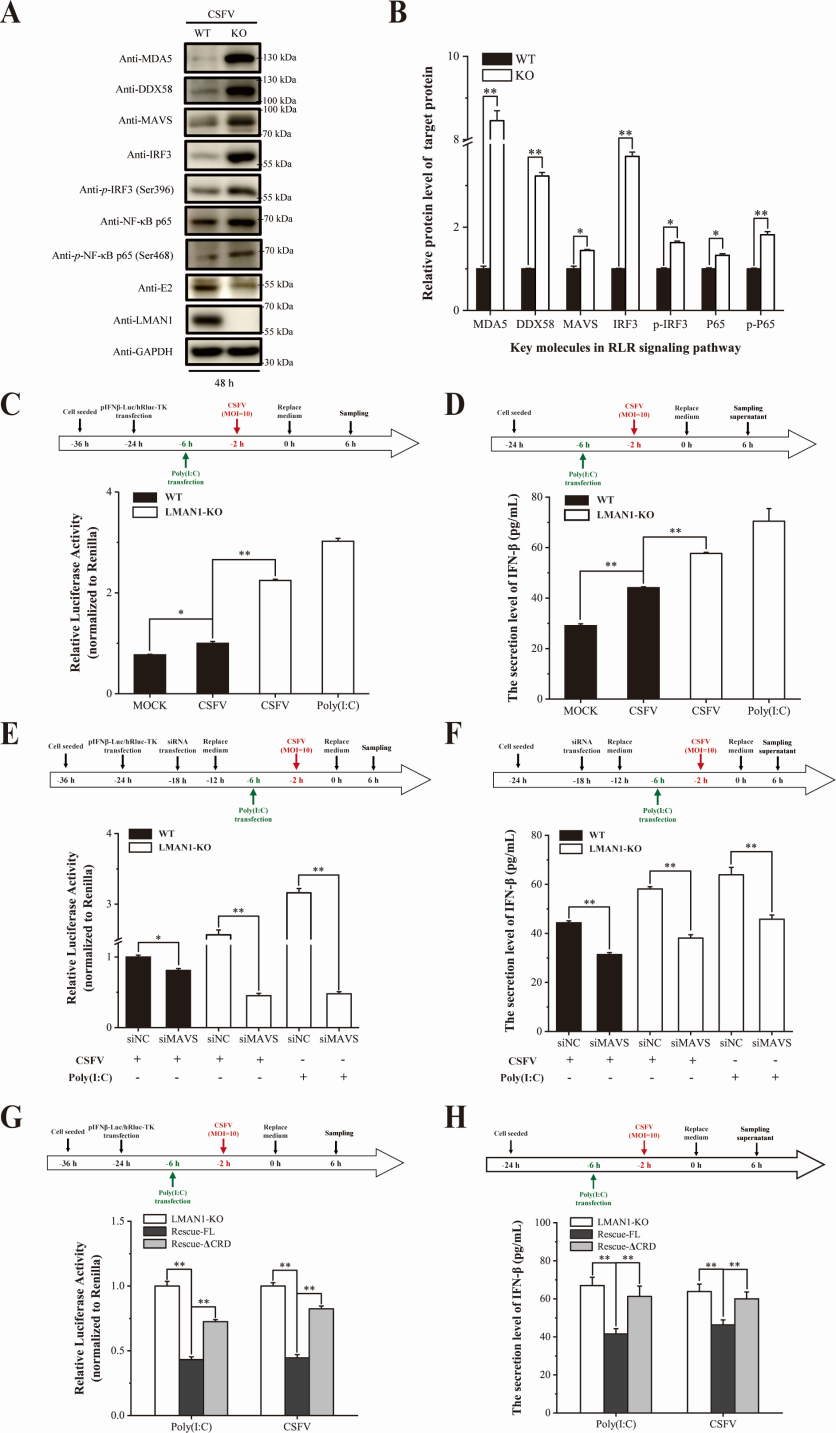
LMAN1 deficiency enhances RLR signaling activation and antiviral responses during CSFV infection. (**A and B**) Protein levels of key RLR-pathway signaling molecules measured by western blot. Loss of LMAN1 markedly increased the protein abundance of sensor proteins (MDA5, DDX58), adaptor (MAVS), and effectors (IRF3, NF-κB p65), as well as the phosphorylation of IRF3 (Ser396) and p65 (Ser468). Band intensities were quantified using ImageJ. (**C**) Antiviral signaling activation monitored by an IFN-β promoter-mediated dual-luciferase reporter assay in WT and LMAN1-KO cells. (**D**) Evaluation of IFN-β production in WT and LMAN1-KO cells with ELISA. (**E and F**) Evaluation of antiviral signaling activity upon reduction of MAVS in LMAN1-KO cells. MAVS knockdown fully rescued the hyperactivation of RLR signaling in LMAN1-deficient cells, indicating that LMAN1 modulates antiviral responses via the MAVS signaling pathway. (**G and H**) The dependency of the CRD domain in suppressing excessive antiviral responses monitored by luciferase reporter assay and IFN-β production. Reintroduced full-length LMAN1, but not ΔCRD, suppressed the hyperactivation of antiviral responses caused by LMAN1 deficiency. Error bars represent standard deviation (*n* = 3). No significance (ns) means *P* > 0.05; * means 0.01 ≤ *P* < 0.05; ** means *P* < 0.01.

To determine whether LMAN1 regulates innate antiviral signaling during CSFV infection, we analyzed key components of the RIG-I-like receptor (RLR) pathway in WT and LMAN1-KO cells. After 48 h of CSFV infection, KO cells exhibited markedly elevated levels of PRRs protein MDA5, DDX58 (RIG-I), and adaptor protein MAVS compared with WT cells (Fig. 8A and B). Upstream signaling activation was further indicated by increased phosphorylation of IRF3 (Ser396) and NF-κB p65 (Ser468), accompanied by increased total IRF3 and p65 protein abundance. These changes collectively demonstrated that loss of LMAN1 amplifies innate immune activation at multiple nodes of the RLR pathway after CSFV infection.

We next assessed functional antiviral signaling using an IFN-β promoter luciferase reporter system. Upon stimulation with either CSFV or poly(I:C), LMAN1-KO cells displayed significantly enhanced IFN-β promoter activity (Fig. 8C) and the production of IFN-β in comparison to WT controls (Fig. 8D), confirming that LMAN1 deficiency leads to heightened transcriptional activation of antiviral pathways.

MAVS functions as an adaptor protein that bridges the viral RNA sensing signals to the activation of downstream genes (26, 27). To investigate the stage at which LMAN1 regulates the activation of cellular innate immune responses, we checked the role of MAVS in amplification of the interferon response after LMAN1 deficiency. siRNA-mediated knockdown conditions were optimized, which showed an effective knockdown of MAVS, without reduction of cell viability (Fig. S11). In WT cells, MAVS knockdown reduced interferon response after CSFV infection. Importantly, the knockdown of MAVS fully rescued the interferon amplification caused by LMAN1 deficiency, which indicates that LMAN1 modulates innate immunity in a manner dependent on the MAVS signaling pathway and acts upstream of MAVS (Fig. 8E and F).

Finally, rescue experiments were conducted to define the structural requirement of LMAN1 for suppressing excessive antiviral responses. Reintroduction of full-length LMAN1 into KO cells significantly decreased poly(I:C)- or CSFV-induced IFN-β promoter activity and the production of IFN-β, restoring signaling to levels comparable to those in WT cells (Fig. 8G and H). In contrast, the CRD-deleted mutant (ΔCRD), which lacks the C-terminal carbohydrate recognition domain, failed to suppress antiviral signaling in KO cells. These results demonstrate that the CRD domain is also essential for the regulatory function of LMAN1 in modulating RLR-mediated antiviral responses.

Collectively, these findings identify LMAN1 as a negative regulator of RLR signaling during CSFV infection and show that loss of LMAN1 enhances innate antiviral responses through MAVS-dependent mechanisms.

## DISCUSSION

In the present study, we identified the ER-Golgi cargo receptor LMAN1 as a host factor hijacked by CSFV to facilitate viral replication-complex formation, thereby enhancing the viral genome replication, and as a suppressor of host RLR antiviral signaling pathway, expanding the function of LMAN1 from glycoprotein trafficking to virus replication and immune regulation.

LMAN1 has been studied primarily in the context of secretory glycoprotein trafficking, in which it forms a complex with MCFD2 and mediates ER-to-Golgi transport of a restricted set of cargos such as coagulation factors V and VIII (28, 29), the cathepsins C and Z (30, 31), and alpha-1 antitrypsin (32), responsible for correct secretory of these physiologically important proteins (33–35). Beyond its canonical trafficking function, LMAN1 also plays a role in the life cycle of several DNA and RNA viruses. It facilitates HBV progeny virion trafficking and egress, is essential for HCV assembly and release, and is required for the infectivity of arenaviruses, coronaviruses, and filoviruses. These studies revealed that LMAN1 participates in the infection of viruses with its canonical function in trafficking of glycoprotein or composing infectious viral particles. However, in our study, when analyzing the early steps of CSFV infection (Fig. 2H), we surprisingly found the vRNA replication rather than attachment or internalization step was impaired upon depletion of LMAN1. This finding led us to further investigate how this cargo receptor affects vRNA replication of a virus. Therefore, we investigated CSFV proteins that interact with LMAN1 and found the viral RNA polymerase NS5B interacts with LMAN1. This physical interaction suggested LMAN1 may directly regulate vRNA replication. With biochemical assays *in vitro* and functional data in LMAN1-KO cells (Fig. 6), we clearly showed that LMAN1 participates in replication-complex formation and facilitates efficient viral RNA synthesis. These new findings demonstrated the non-canonical function of LMAN1, offering new insights to CSFV replicating mechanisms. Due to technical difficulties, whether LMAN1 also participates in the late steps such as virion assembly and release was not studied in our research, but it is worthy to further explore the role of LMAN1 in these processes to systematically understand its function in CSFV infection.

Beyond its role in viral RNA synthesis, we also reveal the role of LMAN1 in regulation of immune response. CSFV suppresses host immunity for its propagation and transmission, with various mechanisms for immune evasion (36). CSFV proteins such as N^pro^ and E^rns^ could actively interfere and suppress innate immune response of host cells (37–39), and host factors like HDAC3 with PHGDH (40) also modulate immunity for CSFV infection. Our transcriptome analyses demonstrated that LMAN1 deficiency leads to a progressive reshaping of host gene expression during infection. At later time points after CSFV infection, antiviral and stress-response pathways, particularly the RLR-MAVS signaling pathway, were hyperactivated when LMAN1 was absent. By monitoring the amount and phosphorylation activation of the key molecules, we confirmed the function of LMAN1 in modulating the RLR-MAVS antiviral pathway. Coincidentally, the CRD is also required for modulating antiviral immune response, as deletion of the CRD domain abolishes the function of LMAN1 in immune response suppression (Fig. 8G and H). Interestingly, both functions of LMAN1 in CSFV infection require the intact CRD domain, which raises the question of whether there is a potential connection between the dual role of LMAN1 in CSFV infection.

In LMAN1-knockout cells, although CSFV genome replication is impaired, leading to less viral RNA—the inducer of IFN production—the amount of IFN generated in response to infection is unexpectedly enhanced rather than reduced. A plausible explanation linking these two observations is that defective replication-compartment formation in LMAN1-KO cells compromises the virus’s ability to spatially organize and shield its RNA replication intermediates from cytosolic dsRNA sensors. This makes the viral RNA more visible to RIG-I/MDA5, leading to increased activation of the MAVS-dependent signaling pathway, which in turn stimulates IRF3 and NF-κB and elevates IFN response (Fig. 8). Conversely, once RLR signaling becomes hyperactivated, the resulting antiviral response further suppresses viral RNA synthesis and disrupts replication-organelle homeostasis at later time points, creating a feed–forward loop that amplifies the initial replication defect. Notably, our data confirm that this heightened IFN response depends on MAVS (Fig. 8E and F). However, we have not yet tested whether blocking MAVS/IFN signaling rescues replication-complex formation in LMAN1-KO cells. Therefore, based on the present evidence, we propose that the primary defect caused by LMAN1 knockout is the loss of a CRD-dependent, NS5B-associated proviral function necessary for efficient replication-complex assembly. The enhanced RLR-MAVS signaling likely represents a closely linked–and potentially reinforcing–consequence of impaired replication-compartment formation and reduced immune evasion in the absence of LMAN1.

**Fig 9:**
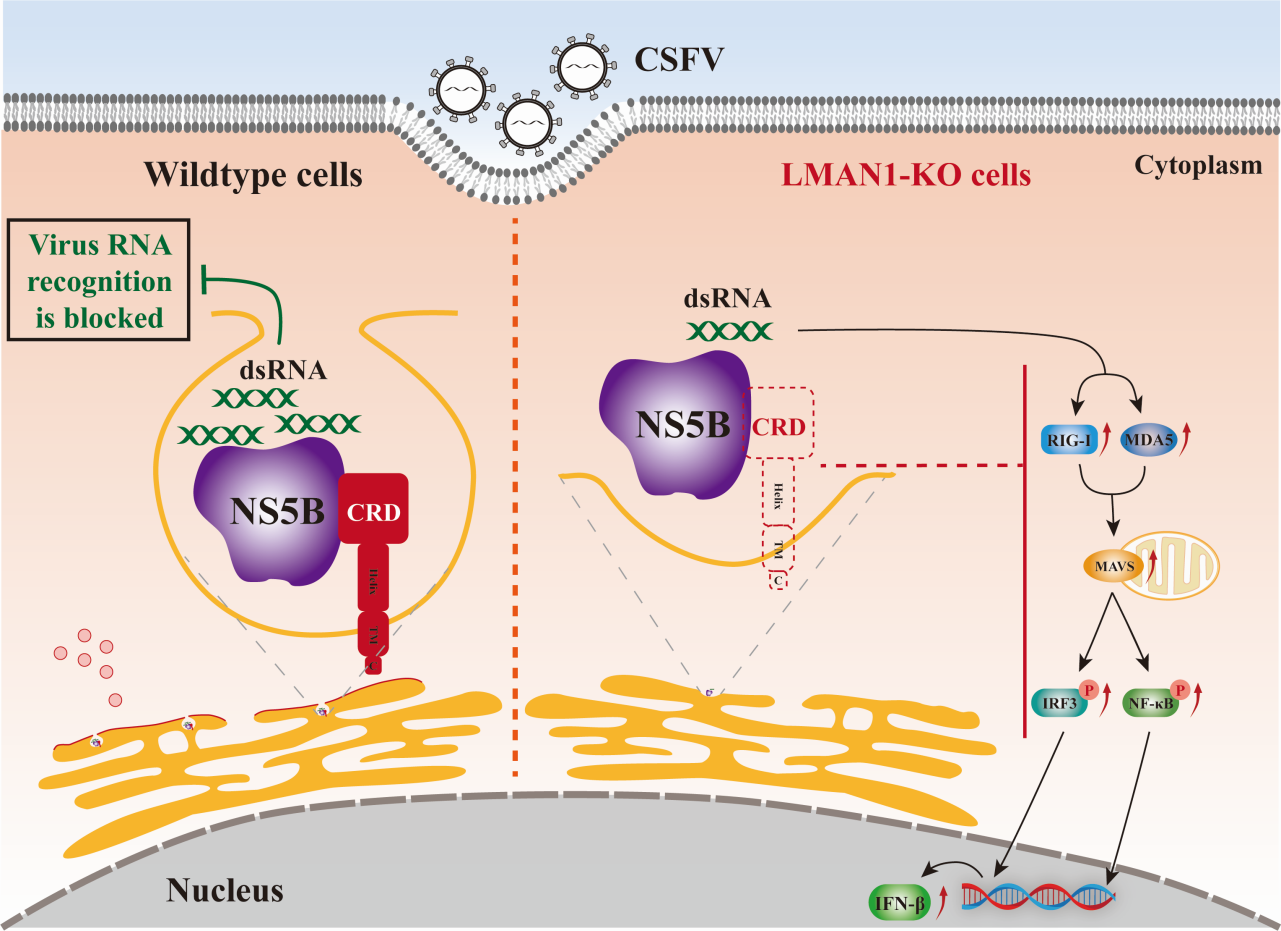
Proposed model illustrating how LMAN1 promotes CSFV replication and modulates RLR-mediated antiviral. LMAN1 interacts with the CSFV RNA-dependent RNA polymerase NS5B through its CRD and participates in viral RNA synthesis in the viral replication factories at ER-associated membranes. Besides, LMAN1 modulates host antiviral responses and suppresses downstream type I IFN-regulated genes via the RLR-MAVS signaling pathway. This model reveals that host LMAN1 promotes the virus infection and proliferation through its dual function during CSFV infection.

In summary, our study identifies LMAN1 as a CRD-dependent, NS5B-interacting host factor that enhances CSFV replication, supports the formation of ER-associated replication complexes, and attenuates RLR-driven antiviral responses (Fig. 9). These findings broaden the functional repertoire of LMAN1 beyond its canonical cargo receptor role and uncover previously unappreciated dual roles in viral RNA synthesis and host immune evasion. Given the evolutionary conservation of LMAN1 and the central importance of ER-derived replication organelles in many positive-sense RNA viruses (41–43), further dissection of the molecular basis of LMAN1 in viral RNA replication and innate immunity regulation may provide a general mechanism for virus infection and host-directed antiviral strategies.

## Data Availability

The data for RNA-seq analysis are openly available in Figshare at 10.6084/m9.figshare.31094431. The rest data analyzed during this study are included in this article and its supplemental material.

## AFTER EPUB

*[This article was published on 3 March 2026 with duplicated text in Materials and Methods. The duplication was removed in the current version, posted on 24 March 2026.]*

## SUPPLEMENTAL MATERIAL

Supplemental material jvi.00069-26-s0001.docx Table S1; Fig. S1 to S11.

ASM does not own the copyrights to Supplemental Material that may be linked to, or accessed through, an article. The authors have granted ASM a non-exclusive, world-wide license to publish the Supplemental Material files. Please contact the corresponding author directly for reuse.
