# Supplementary material for: A dual role for ER-Golgi cargo receptor LMAN1 in supporting CSFV replication and restraining RLR signaling
Source: J Virol. 2026 Mar 3;100(3):e00069-26. doi: 10.1128/jvi.00069-26 (PMC13011374; doi:10.1128/jvi.00069-26)
Supplement: Supplemental material — Table S1; Fig. S1 to S11. [file jvi.00069-26-s0001.docx]

**Supplemental Material**

**Supplementary Table S1 Primers used in this study.**

| Primer name | Sequence (5’ to 3’) | Purpose |
| --- | --- | --- |
| LMAN1-EGFP-F | CGCCTCGAGATGGCGAGATCCAGGCA | Full cDNA |
| LMAN1-EGFP-R | CGCGGATCCGCAAAGAATTTTTTGGCAGCTG | Full cDNA |
| LMAN1-Flag-F | CGCGCTAGCGCCACCATGGCGAGATCCAGGCA | Full cDNA |
| LMAN1-Flag-R | CGCCTCGAGAAAGAATTTTTTGGCAGCTG | Full cDNA |
| FL-F | AGGGAGACCCAAGCTGGCTAGCGCCACCATGGCGAGATCCAGGCA | Mutant |
| FL-R | TAAACGGGCCCTCTAGACTCGAGTCAAAAGAATTTTTTGGC | Mutant |
| Flag-F | GGCGATTACAAGGATGACGACGATAAGGACGGCGTGGGAGGCGACCCCGCGGCTGCTG | Mutant |
| Flag-R | CCCACGCCGTCCTTATCGTCGTCATCCTTGTAATCGCCCCCAACGAACCGAAAGAGCG | Mutant |
| ΔKKAA-R | TAAACGGGCCCTCTAGACTCGAGTCACGCTGCTTTTTTGGCAGCTG | Mutant |
| Δvector-R | TAAACGGGCCCTCTAGACTCGAGTCACTTATCGTCGTCATCCTTGTAATC | Mutant |
| ΔCRD-F | GCTCCACGGGCTCCTCAGCAGCGCAGCCAGGGAAAGAGCCACCTACACCAGATAAAGA | Mutant |
| ΔCRD-R | TCTTTATCTGGTGTAGGTGGCTCTTTCCCTGGCTGCGCTGCTGAGGAGCCCGTGGAGC | Mutant |
| ΔHelix-F | CTTTTCTGACTTTCCAATTGACTGAGCCTATGCCATCAAATGAAAAGCCAAAATGCCC | Mutant |
| ΔHelix-R | GGGCATTTTGGCTTTTCATTTGATGGCATAGGCTCAGTCAATTGGAAAGTCAGAAAAG | Mutant |
| ΔN164A-F | GTTGGAATATTTTTTGATTCTTTTGACGCTGATGGAAAGAAAAATAAT | Mutant |
| ΔN164A-R | ATTATTTTTCTTTCCATCAGCGTCAAAAGAATCAAAAAATATTCCAAC | Mutant |
| shN-F | GATCCGCTTAAACGCATAGTAGGACTCAAGAGAGTCCTACTATGCGTTTAAGCTTTTTG | Knockdown |
| shN-R | AATTCAAAAAGCTTAAACGCATAGTAGGACTCTCTTGAGTCCTACTATGCGTTTAAGCG | Knockdown |
| shLMAN1-1-F | GATCCGGGAATGCTATTCCAAGTTCACAAGAGTGAACTTGGAATAGCATTCCCTTTTTG | Knockdown |
| shLMAN1-1-R | AATTCAAAAAGGGAATGCTATTCCAAGTTCACTCTTGTGAACTTGGAATAGCATTCCCG | Knockdown |
| shLMAN1-2-F | GATCCGCAGCTGAACCGACAGTTAGACAAGAGTCTAACTGTCGGTTCAGCTGCTTTTTG | Knockdown |
| shLMAN1-2-R | AATTCAAAAAGCAGCTGAACCGACAGTTAGACTCTTGTCTAACTGTCGGTTCAGCTGCG | Knockdown |
| shLMAN1-3-F | GATCCGCGGGACATAGATCACTTAGTCAAGAGACTAAGTGATCTATGTCCCGCTTTTTG | Knockdown |
| shLMAN1-3-R | AATTCAAAAAGCGGGACATAGATCACTTAGTCTCTTGACTAAGTGATCTATGTCCCGCG | Knockdown |
| Flag-LMAN1-F | CGCTCTAGAATGGCGAGATCCAGGCAAAGGGGT | Overexpression |
| Flag-LMAN1-R | CGCGCGGCCGCTCAAAAGAATTTTTTGGCAGCTG | Overexpression |
| Vector-Con-R | CGCGCGGCCGCTCACTTATCGTCGTCATCCTTGTAATCGCCCCCAACGAACCGAAAGA | Overexpression |
| LMAN1-KO-F | CACCGCTGTCGCTCTTTCGGTTCGTTGG | Knockout |
| LMAN1-KO-R | AAACCCAACGAACCGAAAGAGCGACAGC | Knockout |
| Rescue-FL(ΔCRD)-F | CAATTGTCAGATCCGCTAGCATGGCGAGATCCAGGCA | CRISPR-resistant |
| Rescue-FL(ΔCRD)-R | CTCCTAGGCGTACGGGATCCTCAAAAGAATTTTTTGGC | CRISPR-resistant |
| Rescue-synonyms-F | TCTGCGCCTTGCTGTTGTCCCTTTTCAGATTCGTCGGGGGCGATTACAAGGATGACGAC | CRISPR-resistant |
| Rescue-synonyms-R | GTCGTCATCCTTGTAATCGCCCCCGACGAATCTGAAAAGGGACAACAGCAAGGCGCAGA | CRISPR-resistant |
| siLMAN1-sense | GGGCAGAAUCGUAUCCAUC(dT)(dT) | siRNA |
| siLMAN1-antisense | GAUGGAUACGAUUCUGCCC(dT)(dT) | siRNA |
| siMAVS-sense | GACAAGACUUAUCAGUAUAUC(dT)(dT) | siRNA |
| siMAVS-antisense | UAUACUGAUAAGUCUUGUCCU(dT)(dT) | siRNA |
| qCSFV-NS5B-F | CCTGAGGACCAAACACATGT | RT-qPCR |
| qCSFV-NS5B-R | TGGTGGAAGTTGGTTGTGTCTG | RT-qPCR |
| qLMAN1-F | GGCCTTTGAGAACTGGGAAG | RT-qPCR |
| qLMAN1-R | CTCCAGGCCTTGATTTTCCG | RT-qPCR |
| qGAPDH-F | TGGAGTCCACTGGTGTCTTCAC | RT-qPCR |
| qGAPDH-R | TTCACGCCCATCACAAACA | RT-qPCR |
| qMAVS-F | CTTACCTGTCCTGCCTCACA | RT-qPCR |
| qMAVS-R | CTGGTAGACACGGGACACTT | RT-qPCR |

| **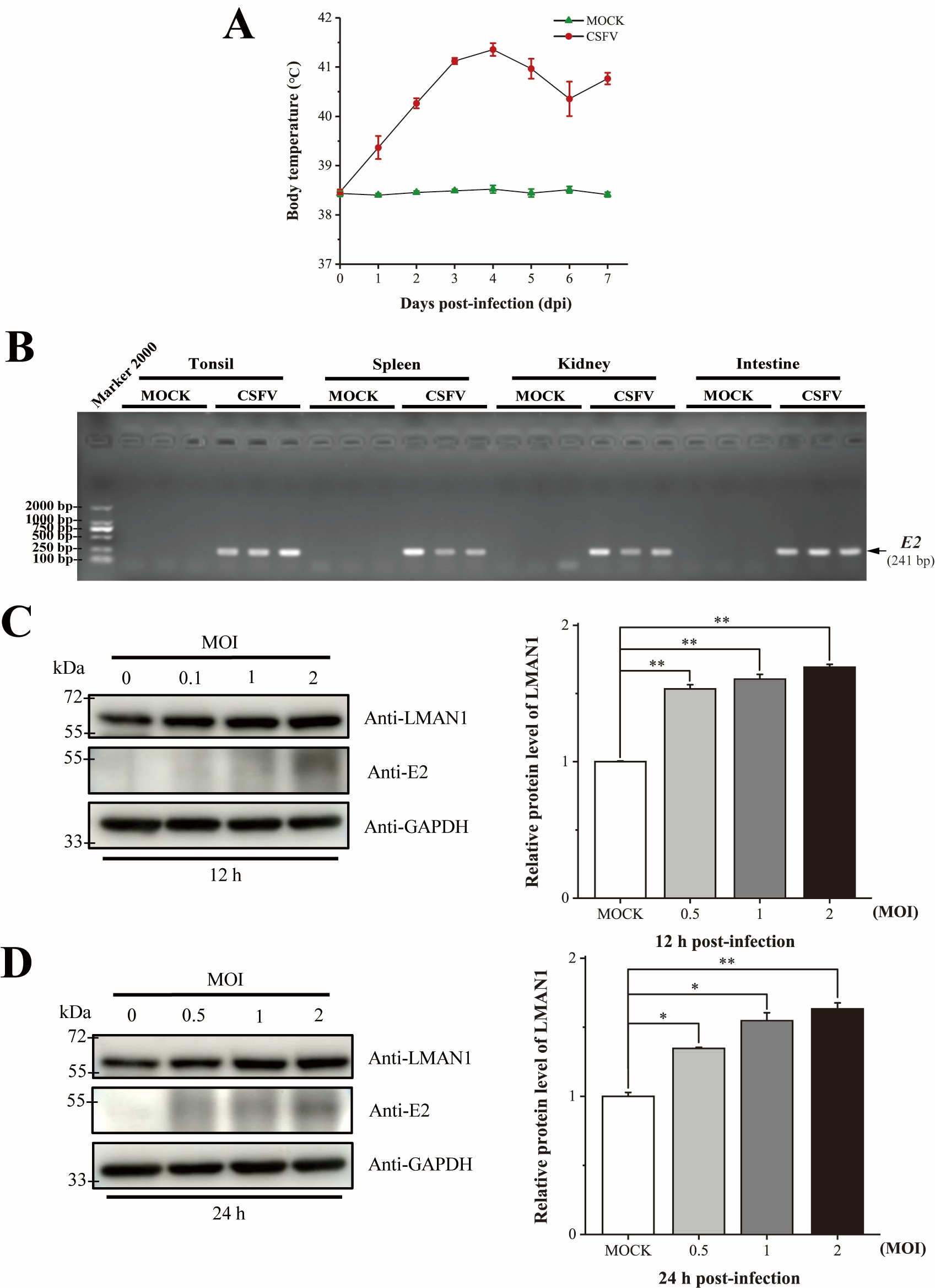** |
| --- |

**Figure S1. Verification of CSFV infection in pigs and modulation of LMAN1 expression.**

(A) Dynamic changes in rectal temperature of piglets following CSFV infection over 7 days post-infection (dpi).

(B) Confirmation of CSFV infection by detection of CSFV E2 structural genes in multiple tissues using reverse transcriptional- PCR. The expected product (size 241 bp, black arrow) was assessed by 1% agarose gel electrophoresis.

(C-D) PK-15 cells were infected with CSFV (MOI = 0.5, 1, 2) for 12 and 24 h. The protein levels of LMAN1, E2 and GAPDH (loading control) were assessed by western blot. Band intensities were quantified using ImageJ.

The error bars represent standard deviation (n=3). No significance (ns) means *p* > 0.05, * means 0.01 ≤ *p* < 0.05, ** means *p* < 0.01 (two-way ANOVA).

| **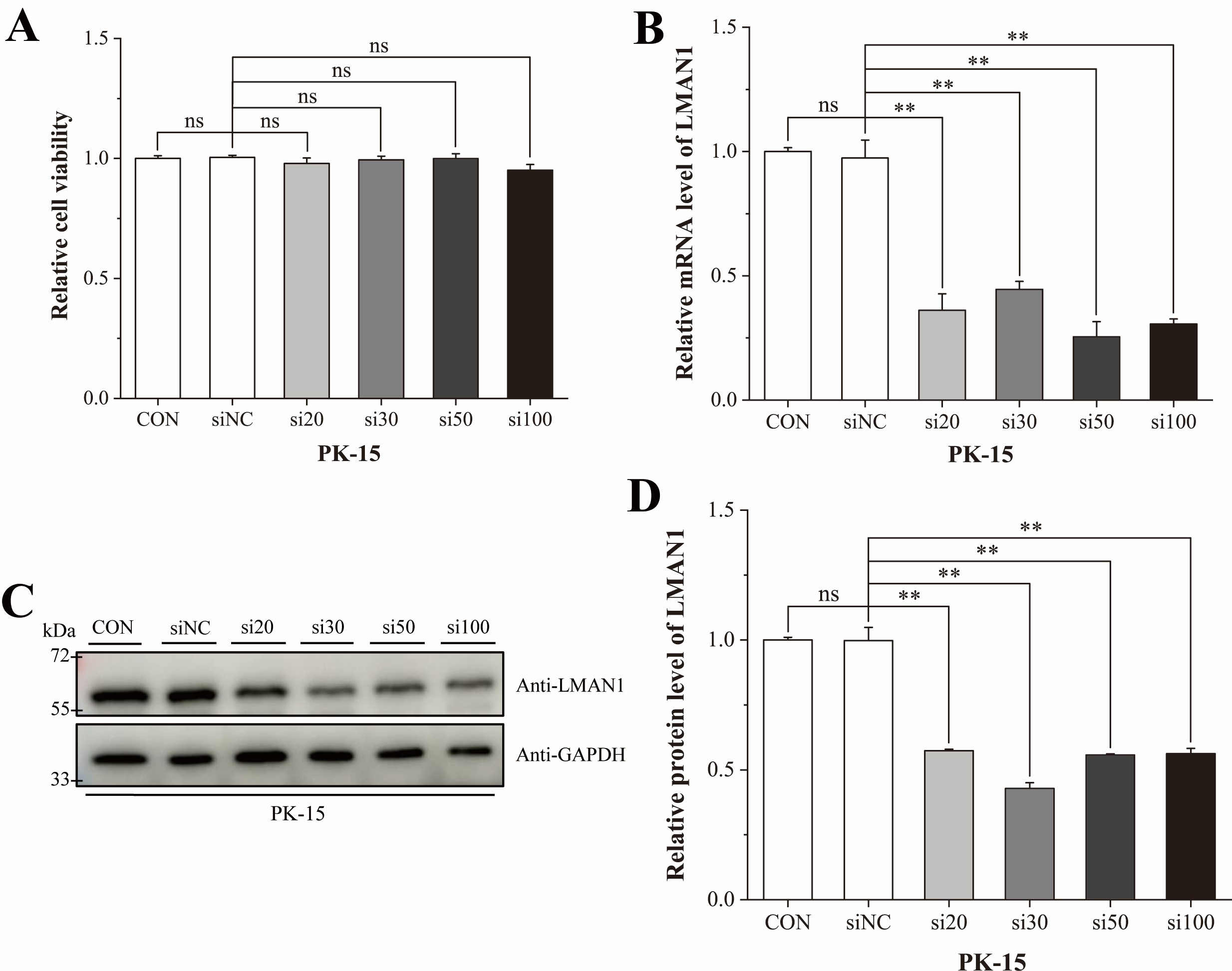** |
| --- |

**Figure S2. Validation of siLMAN1 in PK-15 cells.**

(A-D) PK-15 cells were transfected with LMAN1-specific siRNAs at the indicated concentrations (20, 30, 50 and 100 nM) for 24 h.

(A) Relative cell viability of PK-15 cells following transfection with siLMAN1 or siNC for 24 h, showing that LMAN1 knockdown does not significantly affect cell viability.

(B-D) LMAN1 knockdown efficiency was evaluated by RT-qPCR and western blot, with untreated PK-15 cells and cells transfected with a non-targeting siRNA (siNC) as controls. Band intensities were quantified using ImageJ. A concentration of 30 nM siLMAN1 produced the most efficient knockdown and was used in subsequent experiments.

The error bars represent standard deviation (n=3). No significance (ns) means *p* > 0.05, * means 0.01 ≤ *p* < 0.05, ** means *p* < 0.01 (two-way ANOVA).

| **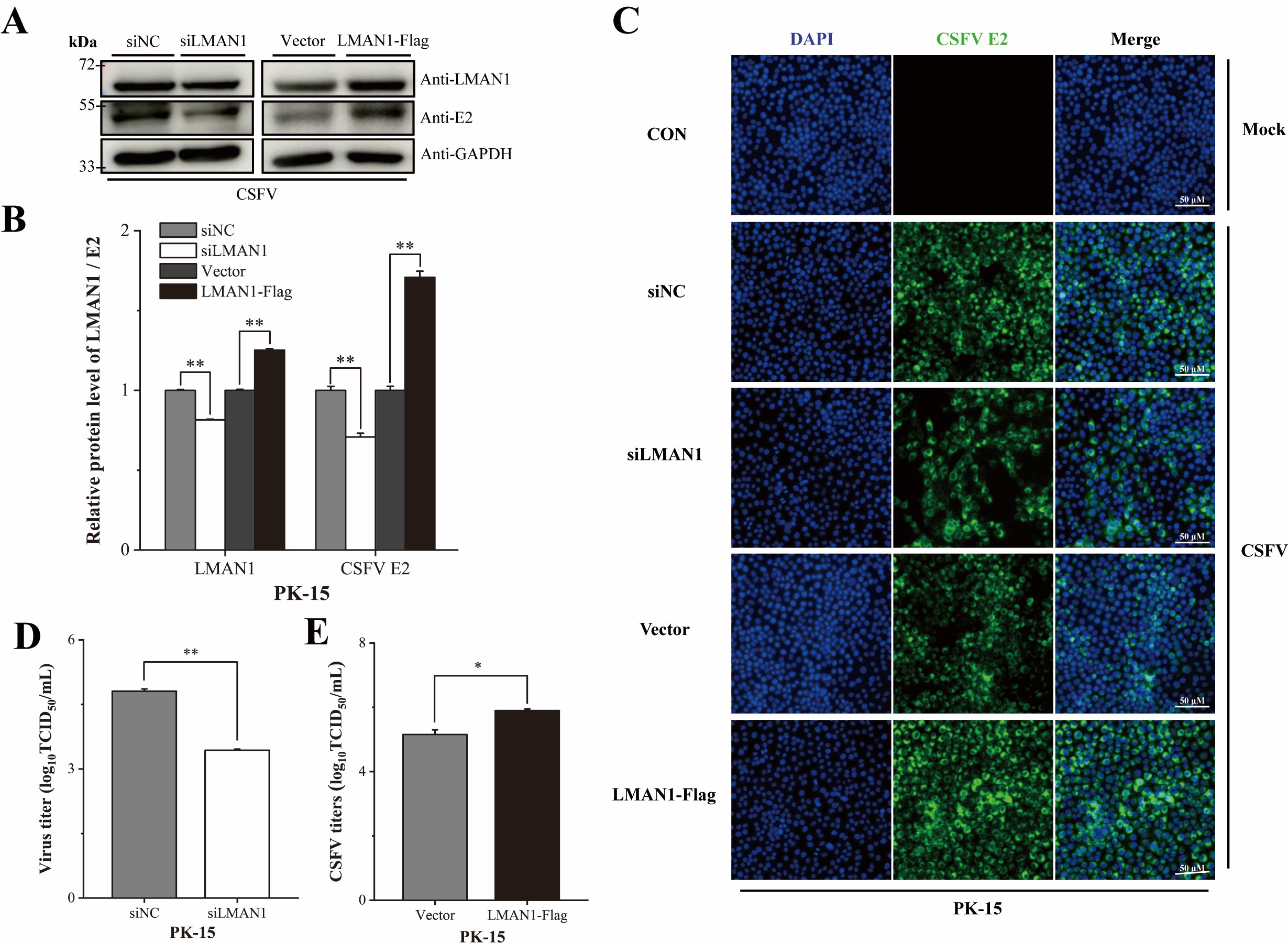** |
| --- |

**Figure S3. LMAN1 expression is required for efficient CSFV infection.**

(A-B) PK-15 cells were transfected with siLMAN1 or siNC for 24 h and then infected with CSFV (MOI = 1) for 48 h, or transfected with LMAN1-Flag or empty vector for 24 h and then infected with CSFV (MOI = 1) for 48 h. Protein levels of LMAN1, CSFV E2, and GAPDH (loading control) were determined by western blot.

(C) PK-15 cells were transfected with siLMAN1 or siNC for 24 h and infected with CSFV (MOI = 10) for 24 h, or transfected with LMAN1-Flag or empty vector for 24 h and infected with CSFV (MOI = 10) for 24 h. Cells were fixed and subjected to immunofluorescence staining using a mouse anti-CSFV E2 antibody. Nuclei were counterstained with DAPI. Scale bars = 100 μm.

(D-E) PK-15 cells were transfected with siLMAN1 or siNC for 24 h and infected with CSFV (MOI = 1) for 24 h, or transfected with LMAN1-Flag or empty vector for 24 h and infected with CSFV (MOI = 1) for 24 h. CSFV titers were determined by a TCID_50_ assay based on immunofluorescence detection.

The error bars represent standard deviation (n=3). No significance (ns) means *p* > 0.05, * means 0.01 ≤ *p* < 0.05, ** means *p* < 0.01 (two-way ANOVA).

| **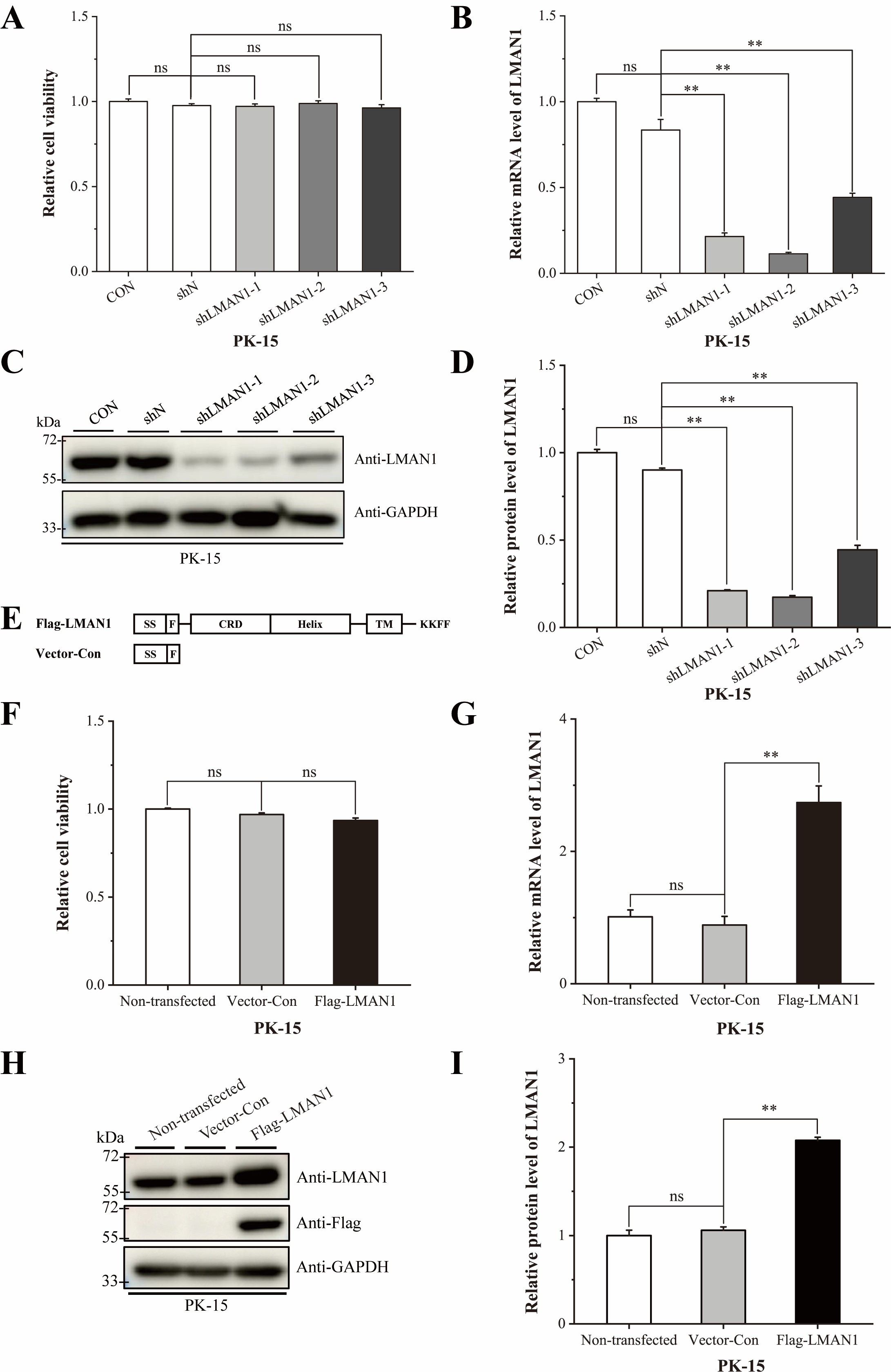** |
| --- |

**Figure S4. Generation and identification of PK-15 cell lines with stable LMAN1 knockdown and overexpression.**

(A-D) Characterization of LMAN1 knockdown cell lines.

(A) Assessment of cell viability following LMAN1 knockdown. The cell viability of the knockdown cell line was detected using the CCK-8 kit, which demonstrated that silencing the LMAN1 protein had non-effect on cell viability. the cell lines of LMAN1 knockdown can be utilized for subsequent experiments.

(B-D) Validation of LMAN1 knockdown by RT-qPCR and western blot. Cell samples from the knockdown cell lines were analyzed for LMAN1 expression, PK-15 cells were used as a control, and PK-15 cells transduced with a non-targeting shRNA plasmid (shN) served as a negative control. Band intensities were quantified using ImageJ. Among the constructs tested, shLMAN1-2 produced the most pronounced reduction in LMAN1 expression and was selected for subsequent experiments.

(E-I) Characterization of LMAN1 overexpression cell lines.

(E) Schematic representation of LMAN1 expression constructs. SS, signal sequence; F, Flag epitope tag; CRD, carbohydrate recognition domain; Helix, helical or stalk domain; TM, transmembrane. Flag-LMAN1 encodes full-length LMAN1 with a Flag-tag, whereas Vector-Con encodes only the SS fused to a Flag-tag and serves as a vector control.

(F) Assessment of cell viability following LMAN1 overexpression. The cell viability of the overexpression cell line was detected using the CCK-8 kit, which demonstrated that ectopic expression of LMAN1 protein had non-effect on cell viability. the cell lines of LMAN1 overexpression can be utilized for subsequent experiments.

(G-I) Validation of LMAN1 overexpression by RT-qPCR and western blot. Using PK-15 cells as a control and CMV-ΔVector-expressing cells as a negative control, protein samples from the overexpression cell lines were analyzed for LMAN1 expression. Band intensities were quantified using ImageJ. LMAN1 expression in the CMV-FL cell line was significantly higher than in control cells, confirming successful generation of the LMAN1 overexpression cell line.

The error bars represent standard deviation (n=3). No significance (ns) means *p* > 0.05, * means 0.01 ≤ *p* < 0.05, ** means *p* < 0.01 (two-way ANOVA).

| **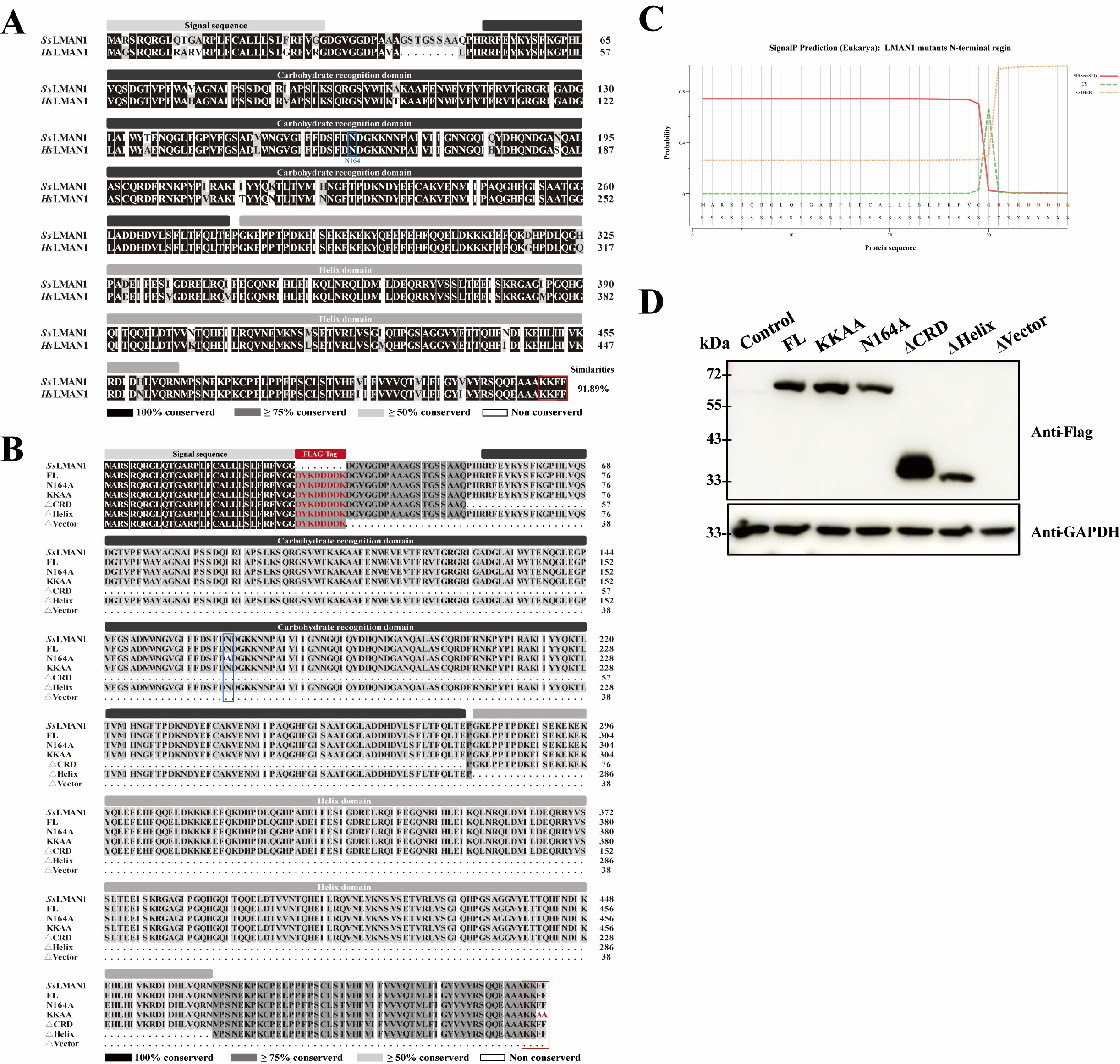** |
| --- |

**Figure S5. Design and characterization of LMAN1 mutants.**

(A) Multiple sequence alignment of *Sus scrofa* LMAN1 (*Ss*LMAN1) and *Homo sapiens* LMAN1 (*Hs*LMAN1), highlighting the N-terminal signal sequence, carbohydrate recognition domain, Helix domain, and the C-terminal KKFF retrieval motif. The N164 sites are shown in box. Residues with different degrees of conserved are shaded in different colors.

(B) Alignment of SsLMAN1 full-length (FL) and mutant sequences (N164A, KKAA, ΔCRD, ΔHelix, and ΔVector), indicating the positions of the N164 residue within the CRD, the helix domain, and the KKFF motif mutated to KKAA.

(C) SignaIP prediction of the N-terminal signal peptide of LMAN1 mutants, showing the predicted signal peptide probability and cleavage site.

(D) Immunoblot analysis of Flag-tagged LMAN1 FL and mutant proteins expressed in HEK-293T cells, with GAPDH as control.

| **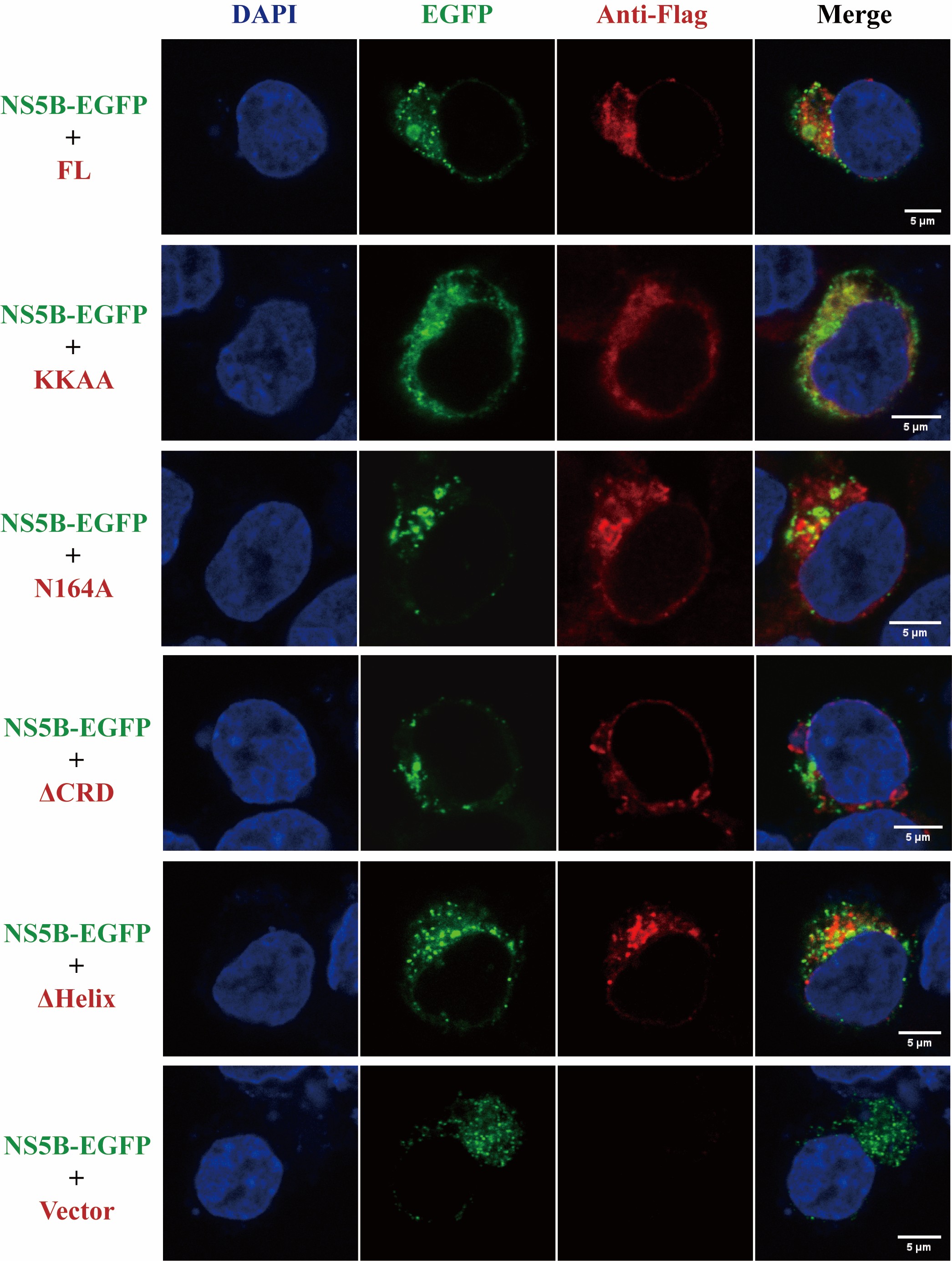** |
| --- |

**Figure S6. Co-localization analysis of LMAN1 mutants with the CSFV NS5B.**

Confocal microscopy of cells co-expressing NS5B-GFP and the indicated LMAN1 constructs, showing nuclear staining (DAPI, blue), NS5B-GFP (green), LMAN1-Flag (red), and merged images to visualize subcellular co-localization. Scale bars = 5 μm.

| 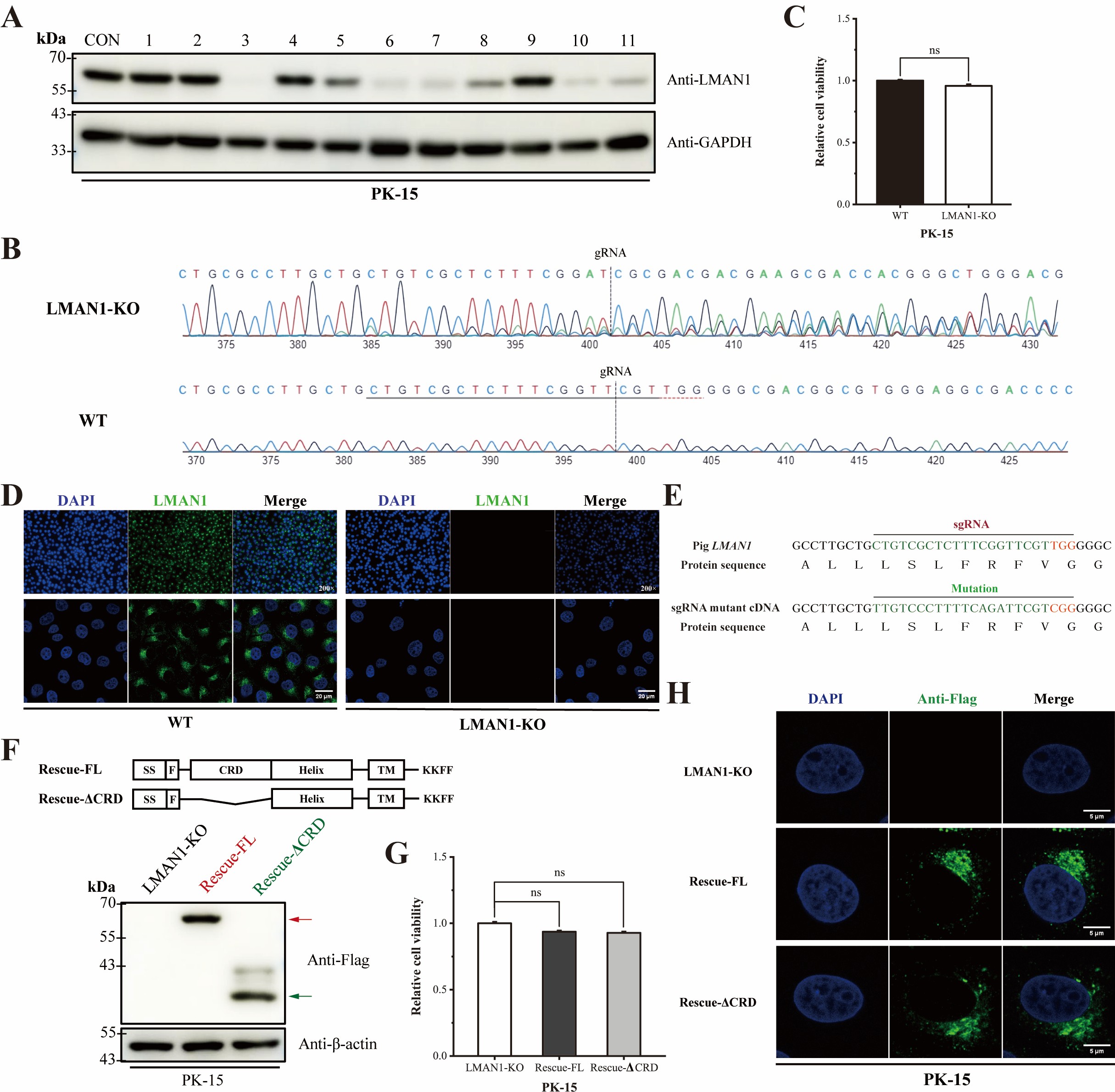 |
| --- |

**Figure S7. Generation and characterization of LMAN1-knockout and -rescue cell lines.**

(A) Immunoblot analysis of endogenous LMAN1 in wild-type (WT) and CRISPR/Cas9-edited LMAN1-knockout (LMAN1-KO) cells. GAPDH serves as the loading control.

(B) After sequencing the genomes of WT and LMAN1-KO cells, the sequence information was analyzed using EZ-editor™ Gene Editing Tool (<https://www.rc-crispr.com/tools/gas.html>).

(C) Relative cell viability of WT and LMAN1-KO cells determined by CCK-8 kit.

(D) Immunofluorescence staining of LMAN1 (green) in WT and LMAN1-KO cells; nuclei are counterstained with DAPI (blue).

(E) Design of CRISPR-resistant LMAN1 cDNA. Perform synonymous mutations on the LMAN1 sgRNA used in the LMAN1-KO cells.

(F) Immunoblot detection of Flag-tagged full-length LMAN1 (Rescue-FL) and the CRD-deleted mutant (Rescue-ΔCRD) expressed in LMAN1-KO cells. β-actin is used as a loading control.

(G) Relative cell viability of LMAN1-KO, Rescue-FL and Rescue-ΔCRD PK-15 cell lines.

(H) Confocal microscopy of LMAN1-KO, Rescue-FL, and Rescue-ΔCRD cells, showing subcellular localization of Flag-tagged LMAN1 constructs (green). Nuclei were counterstained with DAPI. Scale bars = 5 μm.

The error bars represent standard deviation (n=3). No significance (ns) means *p* > 0.05, * means 0.01 ≤ *p* < 0.05, ** means *p* < 0.01 (two-way ANOVA).

| 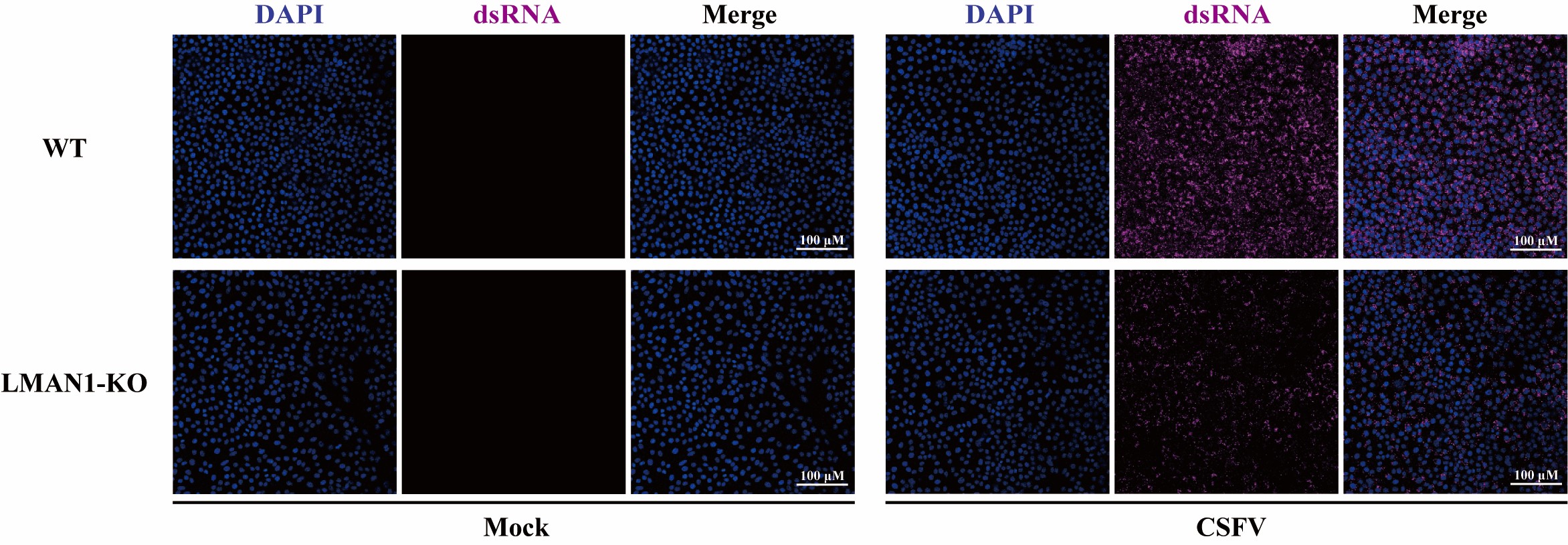 |
| --- |

**Figure S8. LMAN1 deficiency alters viral RNA accumulation.**

WT and LMAN1-KO cells were infected with CSFV (MOI = 20) or DMEM (as a control) for 12 h, then fixed and analyzed by confocal microscopy using mouse anti-dsRNA antibody to visualize viral replication factories. Nuclei were counterstained with DAPI. Scale bars = 100 μm.

| 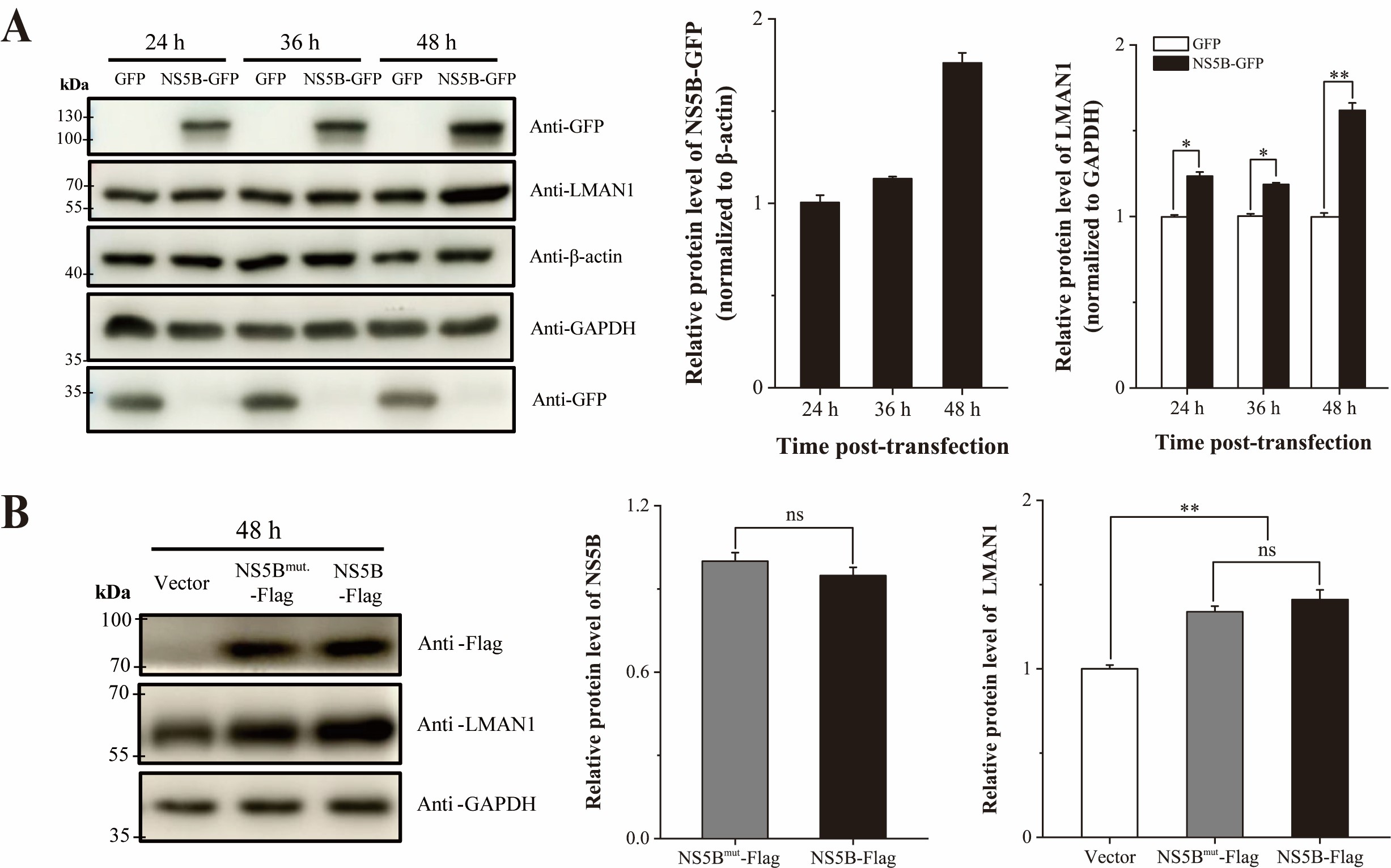 |
| --- |

**Figure S9. The influence of NS5B activity on the expression of LMAN1.**

(A) PK-15 cells were transfected with NS5B-GFP or pEGFP-N1 plasmid for 24 h, 36 h and 48 h, and analyzed by western blot using antibodies against GFP, GAPDH, β-actin, and LMAN1. Band intensities were quantified using ImageJ.

(B) PK-15 cells were transfected with NS5B-Flag, NS5B^mut.^-Flag or Flag-vector (control) for 48 h, and analyzed by western blot with antibodies against Flag, GAPDH, and LMAN1. Band intensities were quantified using ImageJ.

| 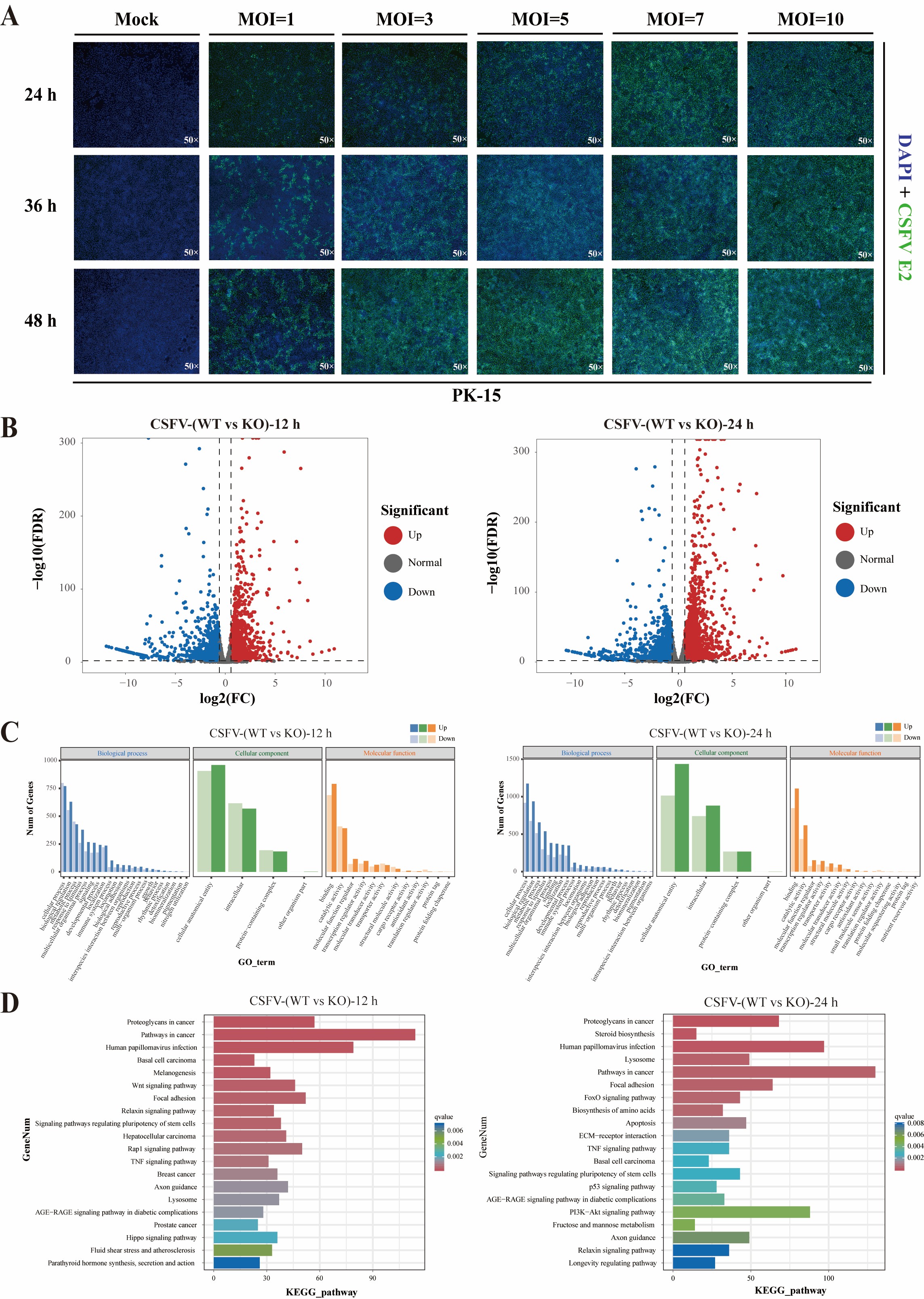 |
| --- |

**Figure S10. Transcriptomic profiling of WT and LMAN1-KO cells following CSFV infection.**

(A) The extent of virus infection in PK-15 cells after CSFV (MOI = 1, 3, 5, 7, 10) infection at 24 h, 36 h and 48 h by immunofluorescence. Viral antigen (CSFV E2) is shown in green and nuclei in blue (DAPI). The results showed that the infection rate of PK-15 cells reached approximately 80%. Therefore, we infected the cells with CSFV (MOI = 10) for transcriptomic profiling.

(B) Volcano plot of DEGs between CSFV-infected WT and LMAN1-KO cells at 12 hpi (left) and 24 hpi (right). Red dots indicate significantly up-regulated genes, blue dots indicate significantly down-regulated genes, and grey dots represent non-significant genes (FDR < 0.05; |log2FC| ≥ 1.5). The vertical and horizontal dashed lines denote the fold-change and FDR thresholds used to define differential expression.

(C) Gene GO enrichment analysis of differentially expressed genes between CSFV-infected WT and LMAN1-KO cells at 12 hpi (left) and 24 hpi (right). Significantly enriched GO terms are shown for the three main categories: Biological Process, Cellular Component and Molecular Function. The intensity of the color represents the up-regulation (deep) and down-regulation (light) of the genes.

(D) KEGG pathway enrichment of DEGs between CSFV-infected WT and LMAN1-KO cells at 12 hpi (left) and 24 hpi (right). The horizontal bar plot shows the top 20 significantly enriched KEGG pathways (adjusted *P* < 0.05). Bar colors represent the adjusted q values, as shown in the scale on the right, with warmer colors indicating higher statistical significance.

| 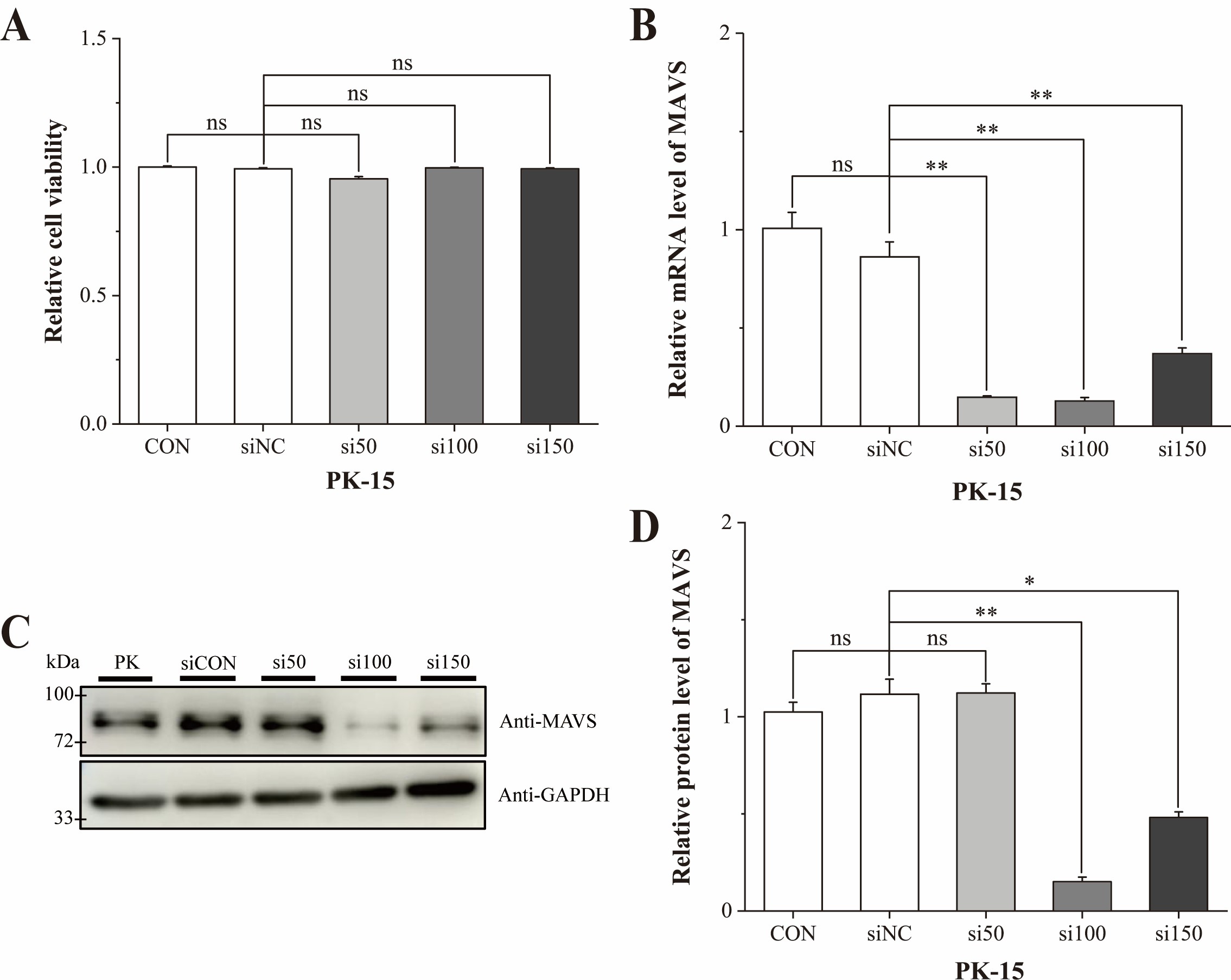 |
| --- |

**Figure S11.** **Validation of siMAVS in PK-15 cells.**

(A-D) PK-15 cells were transfected with MAVS-specific siRNAs at the indicated concentrations (50, 100 and 150 nM) for 24 h.

(A) Relative cell viability of PK-15 cells after transfection with siMAVS or siNC for 24 h, showing that MAVS knockdown does not significantly affect cell viability.

(B-D) MAVS knockdown efficiency was evaluated by RT-qPCR and western blot, with untreated PK-15 cells and cells transfected with a non-targeting siRNA (siNC) as controls. Band intensities were quantified using ImageJ. A concentration of 100 nM siMAVS achieved the most efficient knockdown and was therefore used in subsequent experiments.

The error bars represent standard deviation (n=3). No significance (ns) means *p* > 0.05, * means 0.01 ≤ *p* < 0.05, ** means *p* < 0.01 (two-way ANOVA).
